# Preliminary effects of risk-adapted PSA screening for prostate cancer after integrating PRS-specific and age-specific variation

**DOI:** 10.3389/fgene.2024.1387588

**Published:** 2024-08-01

**Authors:** Xiaomin Liu, Hongyuan Duan, Siwen Liu, Yunmeng Zhang, Yuting Ji, Yacong Zhang, Zhuowei Feng, Jingjing Li, Ya Liu, Ying Gao, Xing Wang, Qing Zhang, Lei Yang, Hongji Dai, Zhangyan Lyu, Fangfang Song, Fengju Song, Yubei Huang

**Affiliations:** ^1^ Department of Epidemiology and Biostatistics, Key Laboratory of Molecular Cancer Epidemiology (Tianjin), National Clinical Research Center for Cancer, Tianjin’s Clinical Research Center for Cancer, Tianjin Medical University Cancer Institute and Hospital, Tianjin, China; ^2^ Department of Epidemiology and Biostatistics, School of Public Health, Tianjin Medical University, Tianjin, China; ^3^ Health Management Center, Tianjin Medical University General Hospital, Tianjin, China; ^4^ Key Laboratory of Carcinogenesis and Translational Research (Ministry of Education), Beijing Office for Cancer Prevention and Control, Peking University Cancer Hospital and Institute, Beijing, China

**Keywords:** prostate cancer, PRS, PSA, screening, age-specific

## Abstract

**Background:**

Although the risk of prostate cancer (PCa) varies across different ages and genetic risks, it’s unclear about the effects of genetic-specific and age-specific prostate-specific antigen (PSA) screening for PCa.

**Methods:**

Weighed and unweighted polygenic risk scores (PRS) were constructed to classify the participants from the PLCO trial into low- or high-PRS groups. The age-specific and PRS-specific cut-off values of PSA for PCa screening were determined with time-dependent receiver-operating-characteristic curves and area-under-curves (tdAUCs). Improved screening strategies integrating PRS-specific and age-specific cut-off values of PSA were compared to traditional PSA screening on accuracy, detection rates of high-grade PCa (Gleason score ≥7), and false positive rate.

**Results:**

Weighted PRS with 80 SNPs significantly associated with PCa was determined as the optimal PRS, with an AUC of 0.631. After stratifying by PRS, the tdAUCs of PSA with a 10-year risk of PCa were 0.818 and 0.816 for low- and high-PRS groups, whereas the cut-off values were 1.42 and 1.62 ng/mL, respectively. After further stratifying by age, the age-specific cut-off values of PSA were relatively lower for low PRS (1.42, 1.65, 1.60, and 2.24 ng/mL for aged <60, 60–64, 65–69, and ≥70 years) than high PRS (1.48, 1.47, 1.89, and 2.72 ng/mL). Further analyses showed an obvious interaction of positive PSA and high PRS on PCa incidence and mortality. Very small difference in PCa risk were observed among subgroups with PSA (−) across different age and PRS, and PCa incidence and mortality with PSA (+) significantly increased as age and PRS, with highest risk for high-PRS/PSA (+) in participants aged ≥70 years [HRs (95%CI): 16.00 (12.62–20.29) and 19.48 (9.26–40.96)]. The recommended screening strategy reduced 12.8% of missed PCa, ensured high specificity, but not caused excessive false positives than traditional PSA screening.

**Conclusion:**

Risk-adapted screening integrating PRS-specific and age-specific cut-off values of PSA would be more effective than traditional PSA screening.

## Introduction

Prostate cancer (PCa) is the most common cancer and the second leading cause of cancer death in men worldwide (Siegel et al., 2023). In addition to efforts on improved treatment and primary prevention of PCa, prostate-specific antigen (PSA) screening has long been recognized as an effective strategy to reduce the burden of PCa and has been widely used in Western countries for decades (Arsov et al., 2022). The European Randomized Study of Screening for Cancer (ERSPC) showed that PSA screening significantly reduced PCa mortality by 25% among men aged 50–74 years (Hugosson et al., 2019). However, since benign prostatic diseases could also present elevated PSA, specificity of PSA for PCa screening is relatively poor and could result in a large number of false positive cases (Nadler et al., 1995; Sindhwani and Wilson, 2005). Previous research showed that only 26% of men with serum PSA levels between 4.1 and 9.9 ng/mL had PCa detected during biopsy (Catalona et al., 2017).

Previous studies also found a clear correlation between PSA and age, and PSA levels increased with age (Babaian et al., 1992). Moreover, Oesterling found that PSA screening with age-specific cut-off values can improve both the sensitivity in young men and the specificity in elderly men (Oesterling et al., 1993). In addition, the risk of PCa varied across different genetic background, and the heritability of PCa was nearly 57% (Mucci et al., 2016). Up until now, genome-wide association studies (GWAS) have identified over 300 genetic variants independently associated with risk of PCa (Benafif et al., 2018; Schumacher et al., 2018; Conti et al., 2021; Plym et al., 2022; Wang et al., 2023), and polygenic risk score (PRS) based on GWAS-identified SNPs is widely used to identify populations at high-risk of PCa (Conti et al., 2021). However, few studies translated this PRS-specific and age-specific variation in both PCa risk and PSA level into different screening recommendations, and few studies had investigated the effects of risk-adapted PSA screening integrating both PRS-specific and age-specific variation.

Therefore, this study first aimed to explore PRS-specific and age-specific cut-off values of PSA for screening PCa and then try to evaluate the preliminary effects of risk-adapted PSA screening integrating both PRS-specific and age-specific variation. Moreover, to simplify and promote the use of PRS in large-scale population-based PCa screening, this study had developed and compared different types of PRS and tried to determine the optimal PRS.

## Materials and methods

### Source of population

Participants in this study were selected from the Prostate, Lung, Colorectal, and Ovarian (PLCO) Cancer Screening Trial (https://cdas.cancer.gov/plco/). Cancer data were collected for each participant in the PLCO trial up to 31 December 2009, and mortality data up to December 2015. The design of the PLCO Cancer Screening Trial has been described previously (Gohagan et al., 2000; Miller et al., 2000; Prorok et al., 2000). In short, 76,683 men aged 55 to 74 were recruited from 10 study centers across the United States between 1993 and 2001. Each institution was approved by its institutional review board, and all participants provided written informed consent. Participants were individually randomly assigned to a control or screening arm, stratified equally by center, age, and sex. Participants assigned to the control arm received usual care, while those assigned to the screening arm were invited to undergo screenings for prostate, lung, colorectal and ovarian cancers outlined in the study protocol. Participants screened for PCa received a PSA test annually for 6 years and a digital rectal exam (DRE) annually for 4 years (Gohagan et al., 2000; Prorok et al., 2000; Roobol, 2009).

In the PLCO trial, there were 53,657 men covered in genome-wide association studies (GWAS). After excluding 1,074 men without a qualified baseline questionnaire, 1,058 men with a history of cancer, 1,504 men with prostatectomy, and 114 men who did not receive any PSA test, a total of 49,907 participants were finally included in this study, including 20,662 participants in the control arm and 29,245 participants in the screening arm (Supplementary Figure S1).

### Follow-up and outcome ascertainment

Men with any PSA >4 ng/mL or any suspicious abnormality in the DRE were considered to be screened positive and were advised to undergo a diagnostic evaluation at the discretion of the patient and their primary physician (Andriole et al., 2005; Miller et al., 2018). Staff at each PLCO study center obtained and recorded medical information related to diagnostic evaluation. PCa cases were identified by the above diagnostic evaluation after positive screening, the Annual Study Update (ASU) form inquiring about cancer diagnosis, and periodic linkage to the National Death Index (NDI) for participants who did not respond to ASU form (Gohagan et al., 2000; Miller et al., 2000; Prorok et al., 2000). All cancer characteristics were documented according to the cancer staging manual issued by the American Joint Committee on Cancer (AJCC). High-grade PCa was defined as Gleason score ≥7.

Deaths were determined primarily by the ASU form and supplemented by periodic linkage with NDI. Once any deaths were notified via the ASU form or NDI, PLCO staff obtained death certificates from state bureaus of vital statistics and collected complete death data coded in line with the ninth edition of International Classification of Diseases (ICD-9). For those whose underlying causes of death were not clearly or accurately recorded on the death certificate, the causes of death were reviewed through the end-point adjudication process.

### Genotype quality control and imputation

Index single-nucleotide polymorphisms (SNPs) associated with PCa were searched from the GWAS catalog (https://www.ebi.ac.uk/gwas/) and previously published GWAS on PCa up until October 2023. The imputed GWAS data of the PLCO trial were obtained from the dbGaP website (https://www.ncbi.nlm.nih.gov/projects/gap/cgi-bin/study.cgi?study_id=phs001286.v1.p1. Requestor: Hongji Dai). The imputation was conducted by the PLCO research team. Despite the relatively comprehensive imputation process, some index SNP information remains unavailable in the imputed dataset. Additionally, not all individuals have complete index SNP information. Unavailable index SNPs in the imputed GWAS dataset were searched for agent SNPs which were in high linkage disequilibrium with index SNPs (R^2^ > 0.6) with the LDtrait Tool (https://ldlink.nih.gov/?tab=home) (Lin et al., 2020; Dixon et al., 2022). After excluding agent SNPs with more than 20% missing value and those in strong linkage disequilibrium with other index SNPs (R^2^ > 0.8) but weakly associated with PCa, a total of 153 SNPs were preliminarily selected (Supplementary Tables S1–3). To mitigate the impact on sample size resulting from the deletion of individuals with any missing index SNPs, further imputation was performed for these missing index SNP information using the major allele in the control group (individuals without cancer). After imputing missing data of index SNP and further excluding SNPs with inconsistent associations with PCa reported from previous GWAS, 102 SNPs were finally included in this study (Supplementary Table S4).

### Assessment of covariates

After informed consent, each participant was provided with a baseline questionnaire to collect demographic information, medical and screening histories reported by the participants. After excluding variables that were not significantly associated with PCa in the univariate analyses, the following potential confounding factors were included in the multivariable analyses: age at entrance (<60, 60–74, 65–69, ≥70 years), race (white, black, other), BMI (0–25, 25–30, >30 kg/m^2^), smoking status (never, current, former), family history of PCa (no, yes), history of previous PSA screening (no, 1 time, ≥2 times), history of enlarged prostate (no, yes), and history of diabetes (no, yes) (Supplementary Table S5).

### Statistical analyses

Chi-square tests were first used to compare the genotype distribution of selected SNPs between PCa cases and controls, logistic regression was used to measure the associations of SNP genotypes and risk allele of selected SNPs with risk of PCa. Unweighted PRS1 and PRS2 were calculated as the sum of a number of risk allele from all 102 SNPs and the SNPs significantly associated with PCa, respectively. Weighted PRS3 and PRS4 were calculated as the sum of risk allele from all 102 SNPs and above validated SNPs weighted with beta of index SNP from univariate logistics regression. The ROC curves of the four PRS were calculated respectively, and the DeLong test was used to make a pairwise comparison between the AUCs. The PRS with the largest AUC was selected as the optimal PRS. According to the cut-off value of PRS based on the ROC curve, the participants was divided into low and high genetic risk groups (low and high PRS).

Time-dependent receiver-operating-characteristic curves (tdROCs) and area under curves (tdAUCs) were performed to determine the optimal cut-off value of PSA with the 10-year risk of PCa. Subgroup analyses were first used to determine the age-specific optimal cut-off value of PSA with the 10-year risk of PCa for participants with different age groups (<60, 60–64, 65–69, and ≥70 years). After stratified by PRS, PRS-specific and age-specific optimal cut-off value of PSA were further determined, and the participants were divided into negative PSA [PSA (−)] or positive PSA [PSA (+)] based on the corresponding cut-off values in overall and subgroup population.

Multivariable Cox regression analyses were performed to assess the independent associations of high PRS and positive PSA on PCa incidence and mortality, after adjusting for all the above confounders. The relative risks were measured as hazard ratio (HR) and 95% confidence intervals (CIs). Subgroup analyses were used to explore the stratified association of positive PSA with PCa incidence and mortality by PRS. Age-stratified interaction analyses based on PRS and PSA were introduced to explore joint association of high PRS and positive PSA on PCa incidence and mortality within the age-specific groups, and low PRS and PSA (−) [low PRS/PSA (−)] within the age-specific group was used as the reference group for the interaction term (4 levels). Further interaction analyses based on PRS, PSA and age were introduced to explore age-specific joint association of high PRS and positive PSA on PCa incidence and mortality across different age groups, and low PRS/PSA (−) in age <60 years was used as the uniform reference group (16 levels).

Based on age-specific and PRS-specific cut-off values of PSA, we proposed three improved PSA screening strategies parallel to traditional PSA screening strategy. For traditional PSA screening, positive PSA was defined as any PSA above diagnostic criteria (>4 ng/mL). For the first improved strategy, positive PSA was defined as greater than age-specific PSA cut-off value. For the second improved strategy, positive PSA was defined as greater than age-specific and PRS-specific PSA cut-off values. To reduce false positive and improve the detection of high-grade PCa cases, based on the second improved strategy, positive PSA in the third improved strategy was defined as PSA greater than age-specific and high-PRS-specific cut-off values after excluding positive PSA in low-PRS group. Compare the screening performance of different screening strategies by calculating sensitivity, specificity, positive predictive value (PPV), negative predictive value (NPV), proportion of high-grade PCa (defined as Gleason score ≥ 7), and false positive proportion of different screening strategies (defined as the number of participants with false positive divided the total number of all participants).

All analyses were performed with R (version 4.1.2) and SPSS (version 25.0). The tdROCs were drawn by the package “riskRegression” (version 2021.10.10). The p-value < 0.05 (two-tailed) was considered statistically significant.

## Results

### Development of PRS and classification of population by PRS

After a median follow-up of 11.6 years, a total of 5,986 PCa cases and 516 PCa deaths were identified among 49,907 participants. There were 2,410 PCa cases and 218 PCa deaths in the control arm, and 3,576 PCa cases and 298 PCa deaths in the screening arm.

Supplementary Table S2 showed the genotypes distribution of all 102 selected SNP between PCa cases and controls. As further shown in Supplementary Table S4, a total of 80 SNPs were validated to be significantly associated with PCa risk with logistics regression analysis. Therefore, these 80 validated SNPs were used to develop the unweighted PRS2 and weighted PRS4. As shown in Supplementary Figure S2, the AUCs of PRS1, PRS2, PRS3 and PRS4 were 0.607, 0.614, 0.630, and 0.631, respectively. After pairwise comparison of these AUCs by DeLong test, PRS4 with the largest AUC was selected as the optimal PRS (Supplementary Table S6).

According to the cut-off value of PRS4 based on the ROC curve, a total of 63.6% (31,717/49,907) of the participants were classified to the low-PRS group, and 36.4% (18,190/49,907) were assigned to the high-PRS group. As shown in Supplementary Table S7, PCa incidence and mortality in the high-PRS group were higher than those in the low-PRS group [(16.38 vs. 7.62 per 1,000 PYs); (0.80 vs. 0.44 per 1,000 PYs)]. After adjusting for confounding factors, compared with the low-PRS group, the risk of PCa incidence and mortality were still significantly higher in the high-PRS group, with HR (95%CI) of 2.15 (2.04–2.26) and 1.85 (1.55–2.19), respectively.

### Effects of PRS-specific PSA screening on PCa incidence and mortality

**FIGURE 1:**
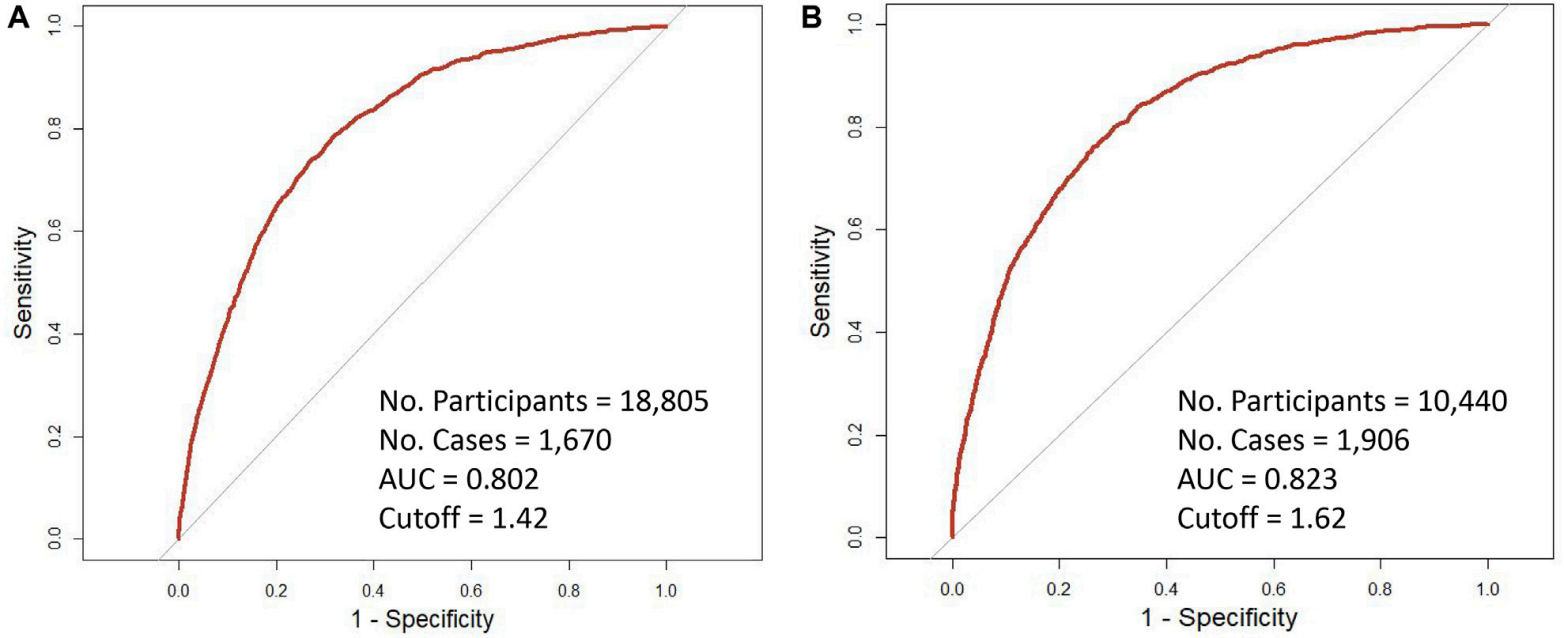
Time-dependent receiver operating characteristic curves of 10-year incidence of prostate cancer with baseline PSA by genetic risk. **(A)** low-PRS; **(B)** high-PRS.

As shown in Figure 1, after stratified by PRS, the tdAUCs were 0.818 and 0.816 for PSA with 10-year risk of PCa for participants with low PRS and high PRS, respectively. Based on the tdROCs, the PRS-specific cut-off values of PSA were 1.42 ng/mL and 1.62 ng/mL for low-PRS and high-PRS participants, respectively. According to the above PRS-specific cut-off values of PSA, a total of 35.7% (6,721/18,805) of low-PRS participants and 39.2% (4,095/10,440) of high-PRS participants were classified as positive PSA.

As shown in Table 1, the risk of both PCa incidence and mortality in participants with positive PSA were higher than those with negative PSA in low-PRS group [(18.33 vs. 2.59 per 1,000 PYs); (0.75 vs. 0.25 per 1,000 PYs)], similar trend was observed for high-PRS group [(41.89 vs. 5.35 per 1,000 PYs); (1.53 vs. 0.40 per 1,000 PYs)]. After adjusting potential confounders, both PCa incidence and mortality were still significantly associated with positive PSA in both low-PRS and high-PRS groups. HRs (95%CI) were 6.26 (5.55–7.05) and 6.15 (5.48–6.90) for PCa incidence in low-PRS and high-PRS groups, respectively, and HR (95%CI) were 1.97 (1.37–2.84) and 2.54 (1.74–3.70) for PCa mortality.

**TABLE 1:** Association of PSA screening with prostate cancer (PCa) incidence and mortality in participants with different genetic risks.

Subgroups	Participants No. (%)	Event No. (%)	Follow-up 1,000 PYs	Event rate, per 1,000 PYs	Unadjusted HR (95%CI)	*p*-value for log-rank test	Adjusted HR (95%CI)^a^	*p*-value^a^
PCa incidence	29,245 (100.0)	3,576 (100.0)	319.80	11.18				
Low PRS								
Negative PSA	12,084 (64.3)	364 (21.8)	140.35	2.59	Ref.	<0.001	Ref.	
Positive PSA	6721 (35.7)	1,306 (78.2)	71.25	18.33	7.08 (6.30-7.95)		6.26 (5.55-7.05)	<0.001
High PRS								
Negative PSA	6345 (60.8)	385 (20.2)	71.90	5.35	Ref.	<0.001	Ref.	
Positive PSA	4,095 (39.2)	1,521 (79.8)	36.31	41.89	7.67 (6.86-8.58)		6.15 (5.48-6.90)	<0.001
PCa mortality	29,245 (100.0)	298 (100.0)	521.12	0.57				
Low PRS								
Negative PSA	12,084 (64.3)	54 (37.8)	216.90	0.25	Ref.	<0.001	Ref.	
Positive PSA	6721 (35.7)	89 (62.2)	118.88	0.75	3.02 (2.15-4.23)		1.97 (1.37-2.84)	<0.001
High PRS								
Negative PSA	6345 (60.8)	45 (29.0)	113.53	0.40	Ref.	<0.001	Ref.	
Positive PSA	4,095 (39.2)	110 (71.0)	71.81	1.53	3.90 (2.76-5.52)		2.54 (1.74-3.70)	<0.001

^a^ Adjusted available variables associated with prostate cancer listed in Supplementary Table S5 and the number of PSA screening and the results of the first round of DRE have also been adjusted, and missing data of each variable were coded as independent group in the multivariable COX regression.

### Determination of age-specific and PRS-specific cut-off values for PSA screening

**FIGURE 2:**
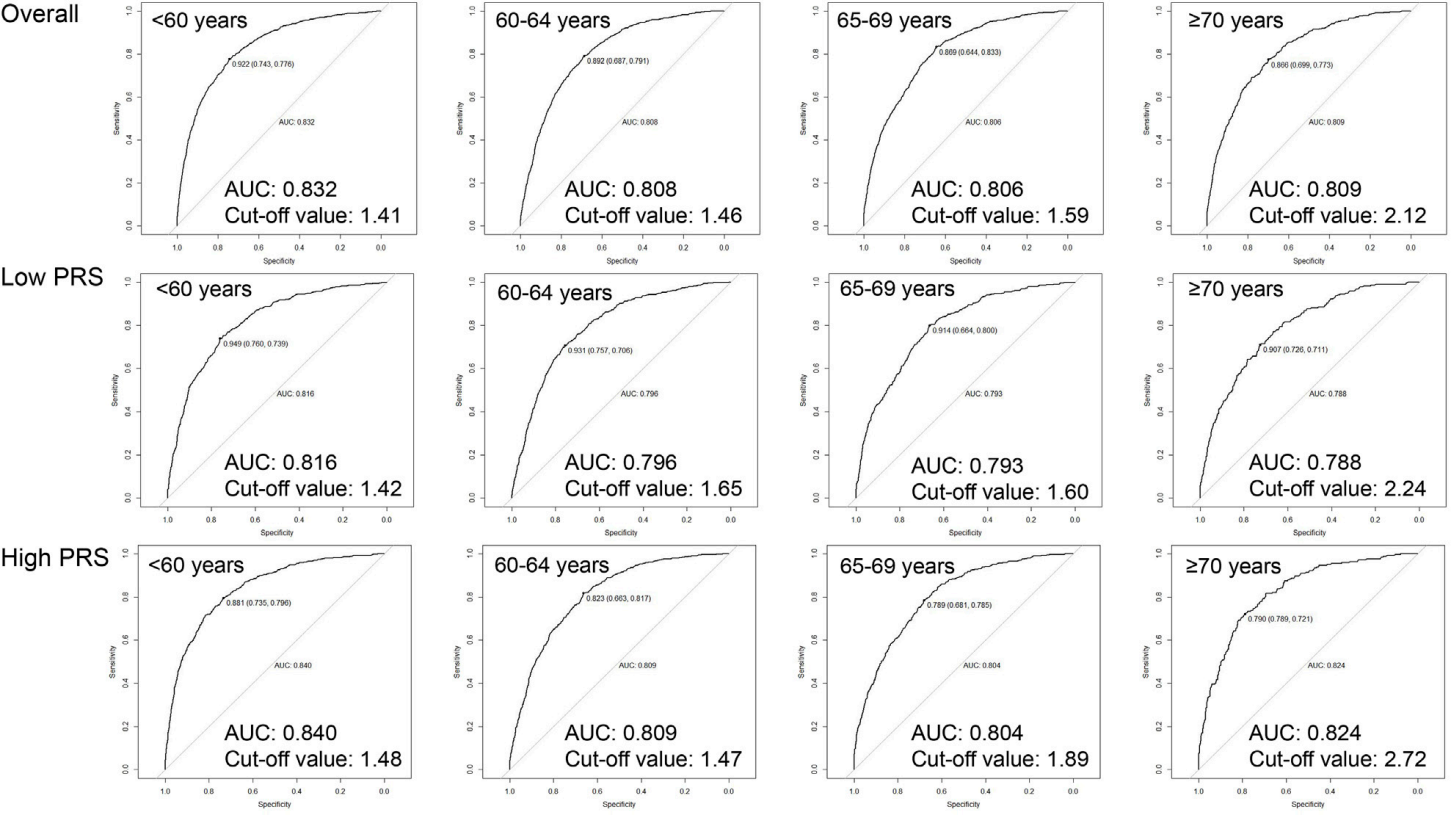
Age-specific cut-off values of baseline PSA for 10-year incidence risk of prostate cancer by genetic risk.

As shown in Figure 2, age-specific tdAUCs of PSA with 10-year incidence risk of PCa in participants aged <60, 60–64, 65–69, and ≥70 years groups were 0.832, 0.808, 0.806, and 0.809, respectively, and age-specific PSA cut-off values were 1.41, 1.46, 1.59, and 2.12 ng/mL, respectively. After stratifying by PRS, the age-specific tdAUCs of PSA were 0.816, 0.796, 0.793, and 0.788 for low-PRS participants aged <60, 60–64, 65–69, and ≥70 years, respectively, and the age-specific PSA cut-off values were 1.42, 1.65, 1.60, and 2.24 ng/mL, respectively. In the high-PRS group, the age-specific tdAUC for participants aged <60, 60–64, 65–69, and ≥70 years groups were 0.840, 0.809, 0.804, and 0.824, respectively, and the age-specific PSA cut-off values were 1.48, 1.47, 1.89, and 2.72 ng/mL. Overall, the cut-off values of PSA increased with age, and the cut-off values of PSA for high-PRS participants were higher than those for low-PRS participants.

### Associations of age-specific and PRS- specific PSA with PCa incidence and mortality

**FIGURE 3:**
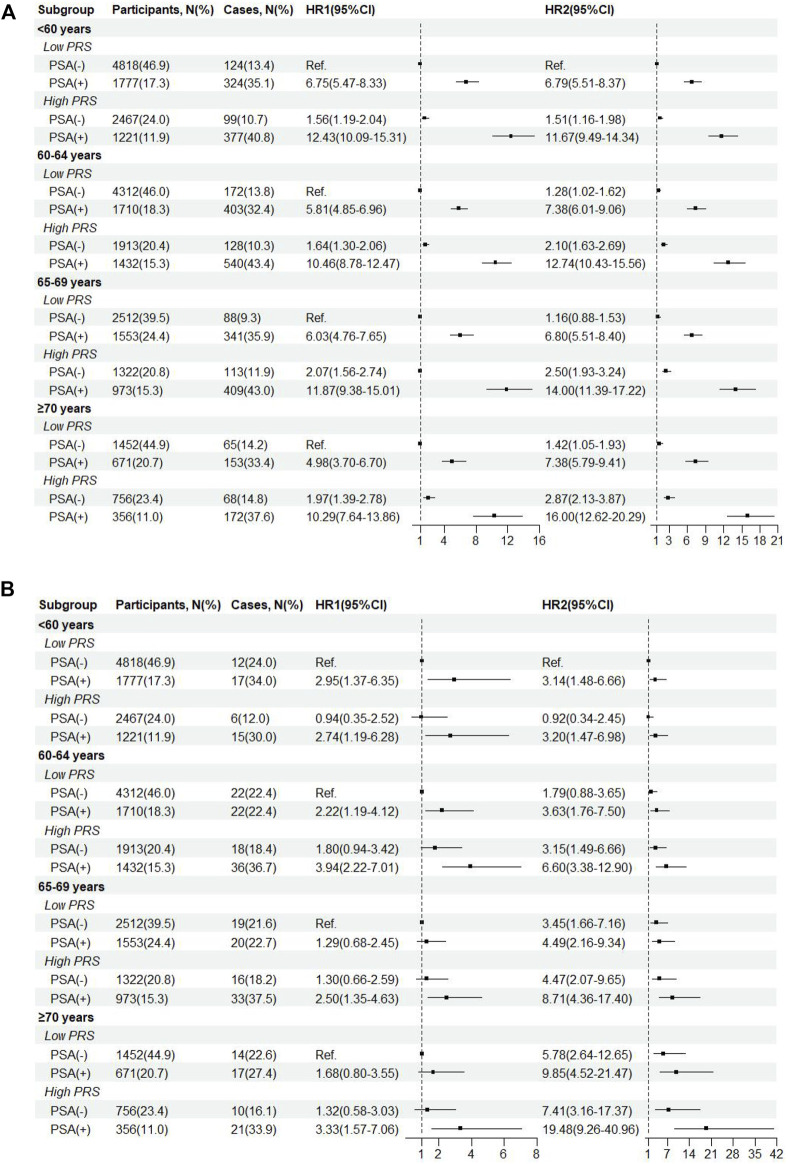
Incidence **(A)** and mortality**(B)** of prostate cancer by age-specific cut-off value of baseline PSA and genetic risk. HR1, HR from age-stratified interaction analyses. HR2, HR from further interaction analyses across different age groups.

As shown in the age-stratified interaction analyses (Figure 3), compared with low PRS/PSA (−) in participants aged <60 years, HR (95%CI) of PCa incidence were 6.75 (5.47–8.33) for low PRS/PSA (+) and 12.43 (10.09–15.31) for high PRS/PSA (+), respectively, while HR (95%CI) of PCa mortality were 2.95 (1.37–6.35) and 2.74 (1.19–6.28). Similar increased risks of both PCa incidence and mortality were consistently observed for both low PRS/PSA (+) and high PRS/PSA (+) compared to low PRS/PSA (−) within other age groups. Further interaction analyses showed very small difference in PCa incidence among all subgroups with PSA (−) across different age and PRS, and risks of PCa incidence and mortality associated with PSA (+) significantly increased as age and PRS. The highest risk of PCa incidence and mortality was found for high-PRS/PSA (+) in participants aged ≥70 years old, with HRs (95%CI) of 16.00 (12.62–20.29) and 19.48 (9.26–40.96), respectively.

### Effects of risk-adapted PSA screening integrating PRS-specific and/or age-specific variation

As shown in Table 2, based on diagnostic criteria of PSA for PCa, the sensitivity, specificity, PPV and NPV of traditional screening strategy were 29.1%, 95.6%, 47.7%, and 90.6%, respectively. Compared with traditional screening strategy, the sensitivity and NPV of the first improved screening strategy increased to 79.2% and 96.0%, expectedly, the specificity and PPV decreased to 70.2% and 27.0%. After further stratification based on age-specific and PRS-specific PSA cut-off values, the sensitivity and NPV of the second improved screening strategy decreased to 76.0% and 95.6%, and the specificity and PPV increased to 72.8% and 28.1%. To further reduce potential false positives and improve the detection of high-grade PCa, after excluding positive PSA participants in the low-PRS group based on the second improved strategy, the third improved strategy showed an increased specificity (90.3%) and PPV (37.6%), while the sensitivity and NPV decreased to 41.9% and 91.8%. To better showed the difference on the specificity between the third improved strategy and the traditional strategy under the given sensitivity, sensitivity analyses were conducted after setting the sensitivity of the traditional strategy to 41.9%. When the sensitivity of the traditional strategy was set to 41.9% (corresponding to the PSA of 2.79 ng/mL), the corresponding specificity was 89.3%, which was still lower than that under the third improved strategy (90.3%).

**TABLE 2:** Evaluation of PSA screening for prostate cancer in populations with different genetic risks.

Methods	Cases	Non-cases	Total	Sensitivity		Specificity		PPV		NPV	
				%	P	%	P	%	P	%	P
Traditional screening strategy (positive screen defined as PSA>4 ng/mL)											
Positive	1,040	1,139	2,179	29.1	Ref.	95.6	Ref.	47.7	Ref.	90.6	Ref.
Negative	2,536	24,530	27,066								
Total	3,576	25,669	29,245								
The first improved screening strategy (positive screen defined as PSA greater than age-specific PSA cutoff values)											
Positive	2,832	7651	10,483	79.2	<0.001	70.2	<0.001	27.0	<0.001	96.0	<0.001
Negative	744	18,018	18,762								
Total	3,576	25,669	29,245								
The sceond improved screening strategy (positive screen defined as PSA greater than age-specific and PRS-specific PSA cutoff values)											
Positive	2,719	6974	9,693	76.0	<0.001	72.8	<0.001	28.1	<0.001	95.6	<0.001
Negative	857	18,695	19,552								
Total	3,576	25,669	29,245								
The third improved screening strategy (positive screen defined as PSA greater than age-specific and high-PRS-specific PSA cutoff values)											
Positive	1,498	2,484	3,982	41.9	<0.001	90.3	<0.001	37.6	<0.001	91.8	<0.001
Negative	2078	23,185	25,263								
Total	3,576	25,669	29,245								

Abbreviations: PPV, positive predictive value; NPV, negative predictive value.

**FIGURE 4:**
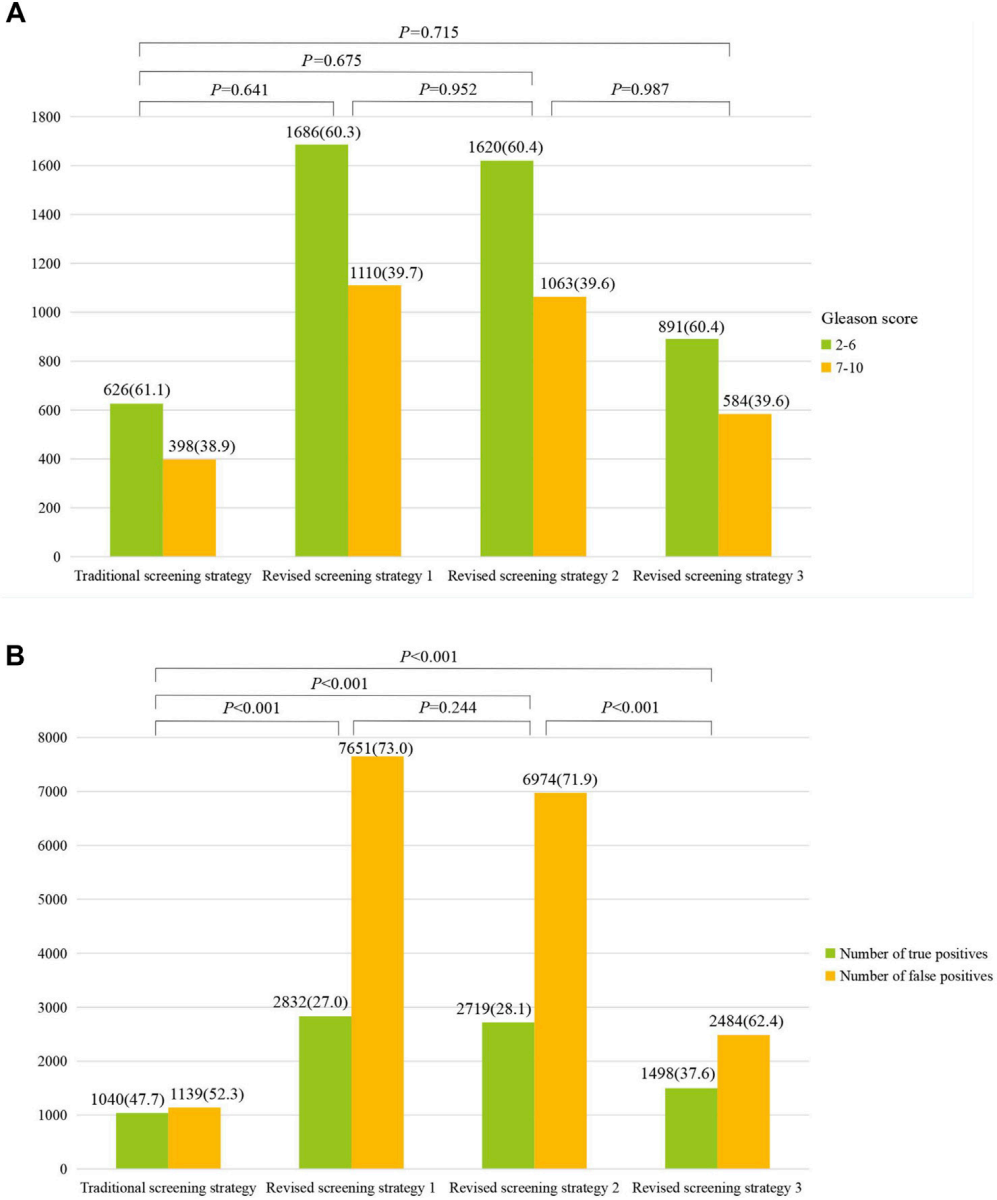
Comparison of high-grade prostate cancer (Gleason score ≥7) **(A)** and proportions of true/false positive **(B)** between different screening strategies.

Further comparison showed that all the detection rates of high-grade PCa from the three improved strategies were higher than that in the traditional strategy (39.7%, 39.6% and 39.6% vs. 38.9%) (Figure 4A), although these differences were not statistically significant. Furthermore, as shown in Figure 4B, the false positive rate of the third improved strategy (62.4%) was significantly lower than those of the first and second revised strategy (73.0% and 71.9%).

## Discussion

In this study, both weighted and unweighted PRSs for PCa were constructed to identify participants at high-risk of PCa. Consistent with previous studies (Pashayan et al., 2015; Schumacher et al., 2018; Seibert et al., 2018; Plym et al., 2022), the risks of PCa incidence and mortality in the high genetic risk group was significantly higher than those in the low genetic risk group. However, to the best of our knowledge, this study is the first study to explore PRS-specific cut-off values of PSA for screening PCa, and the first to further explore the effects of risk-adapted PSA screening integrating both PRS-specific and age-specific variation. Moreover, compared with traditional screening strategy, recommended risk-adapted PSA screening not only reduces missed PCa by 12.8%, but also ensures high specificity, and does not cause excessive false positive.

Previous GWAS had identified 269 SNPs independently associated with PCa risk until now (Conti et al., 2021), and a multiethnic PRS model based on 261 germline SNPs could be used to identify a substantial proportion of men at high risk for PCa (Plym et al., 2022). However, the ability of PRS alone to identify men with PCa was modest, and the AUC of PRS for PCa have not been significantly improved from PRS with top four SNPs to the latest 260-SNP PRS (ranging from 58.5% to 63.7%) (Oh et al., 2020; Sipeky et al., 2020; Kim et al., 2022; Siltari et al., 2023). This small improvement on the AUC suggested that the newly identified SNPs may be useful in exploring the mechanism of PCa incidence and development, however, the contribution of PRS to population-based risk stratification may still derive from the top SNPs. Therefore, it would be more practical to construct a simplified PRS with top SNPs rather than a bloated PRS with increasing number of SNPs before applying PRS to population-based risk stratification or other potential interventions, especially as PRS with large number of SNPs would cost more. This was also why this study aimed to develop different PRS and try to determine the optimal PRS for PCa screening. Although the weighted PRS4 was finally selected as the optimal PRS, the AUC difference between the different PRS was less than 3%. Therefore, when using PRS for population-based risk stratification of PCa, simplified PRS rather than PRS with large number of SNPs should deserve more priority, especially for resources-limited areas.

Most previous population-based PCa screening trials with PSA, including the Cluster Randomized Trial of PSA Testing for Prostate Cancer (CAP), the PLCO trial, the PROBASE trial, the ERSPC, and the GÖTEBORG-2 Trial (Andriole et al., 2009; Arsov et al., 2013; Martin et al., 2018; Hugosson et al., 2019; Hugosson et al., 2022), used a single PSA cut-off value (≥3.0 or 4 ng/mL) as the definition of a positive PSA test and recommended further examinations after positive PSA. Although the European Association of Urology (EAU) recommended the use of PSA testing as part of a risk-adapted strategy for the early detection of PCa in 2021 (Martin et al., 2018), large trials, for example, the STLM3 trial, the PROBASE study, the Göteborg 2 trial, and the ProScreen study (Arsov et al., 2013; Auvinen et al., 2017; Hugosson et al., 2019; Hugosson et al., 2022), had initiated risk-adapted PSA screening since 2012. The common risk-adapted option is to combine PSA with other biochemical indicators, such as the panel of four kallikreins (total PSA, free PSA, intact PSA and human kallikrein-related peptidase-2, hK2), known as the 4Kscore. However, few population-based PCa screening trials adopted risk-adapted PSA screening based on both PRS-specific and age-specific PSA cut-off values, even though age-specific reference ranges for serum PSA had been suggested from 1993 (Oesterling et al., 1993).

Consistent with previous studies (Babaian et al., 1992), this study supported that PSA increased normally with age. Correspondingly, the PSA cut-off values for PCa screening gradually increased with age, which were also similar to previous studies. As in Oesterling and Lankford’s study, the recommended cut-off values of PSA for PCa screening were 3.5, 4.5, and 6.5 ng/mL for men aged 50–59, 60–69, and 70–79, respectively (Oesterling et al., 1993; Lankford et al., 1995). Another multi-ethnic population study showed that the recommended PSA cut-off values for men aged 55–59, 60–64, 65–79, 70–74, and 75–79 years were 3.19, 4.01, 4.90, 5.99, and 7.61 ng/mL, respectively (Matti and Zargar-Shoshtari, 2021). Recent systematic review and meta-analysis including forty-three studies covering 325,514 participants also suggested that the pooled age-specific PSA reference values were 2.1, 3.2, 4.9, and 6.5 ng/mL for men in their 40 s, 50 s, 60 s, and 70 s, respectively (Matti et al., 2022). Notably, the age-specific PSA cut-off values in this study were relatively lower than previous studies. The major reason for the lower PSA cut-off values in this study may be since previous studies were conducted in small sample size of men with some symptoms of prostate (Matti et al., 2022), while this study was conducted in large sample size of community population and most of them did not had any symptoms of prostate. Additionally, as shown in the Supplementary Table S5, more than half of the participants in the PLCO trial had ever received PSA screening before recruitment, which would also lead to lower baseline PSA level and then lead to lower age-specific PSA cut-off values.

Furthermore, we also observed different cut-off values of PSA under different genetic risks, and the PSA cut-off values was higher in the high-PRS group than those in the low-PRS group. Previous studies suggested that not only the PRS of PCa was correlated with serum PSA level (Amin et al., 2015), but also higher PRS of benign prostatic hyperplasia was also correlated with increased PSA level (Gudmundsson et al., 2018). However, few studies have explored PRS-specific cut-off values of PSA in population-based PCa screening, and fewer studies further explored PRS-specific and age-specific cut-off values of PSA for PCa screening. Moreover, several previous studies suggest significantly higher percentage of men had a PSA >4 ng/mL found in high PRS compared to low PRS (Sipeky et al., 2020), and the combination of PRS and PSA (or other indicators including PSA, such as prostate health index) could effectively captured participants at both clinical and genetic high risk of PCa, even in patients with gray-zone PSA (Chou et al., 2022; Ruan et al., 2023). All of these strongly suggested that screening integrating PRS-specific and age-specific cut-off values of PSA would facilitate the identification of men at increased risk for PC.

To improve the effect of PSA screening for PCa, after integrating PRS-specific and/or age-specific cut-off values of PSA, we proposed three improved screening strategies. Expectedly, all of these three screening strategies can significantly improve the sensitivity of PCa and reduce potential missed PCa. The specificity of first and second improved strategies decreased significantly, however, the specificity of the third improved strategy still at a relatively comparable level of traditional screening. Moreover, all three improved strategies detected a higher percentage of high-grade PCa, though none of them were significant. This non-significant improvement suggested that new indicators and/or PSA progression indicators based on multiple PSA tests (such as PSA velocity, PSA doubling time) (Vickers et al., 2009; Orsted et al., 2013; Wallner et al., 2013; Shoaibi et al., 2017; Liu et al., 2023), especially PRS-specific PSA progression indicators, are needed to be proposed to improve early-detection of high-grade PCa in the future. In addition, the third improved strategy improved the detection rate of high-grade PCa without leading to an excessively high false-positive rate. We think that in screening, as many cases as possible should be detected (higher true-positive rate) while trying to avoid overdiagnosis (lower false-positive rate). Detecting as much high-grade PCa as possible might better improve PCa survival while controlling that the false-positive rate would not be too high. Furthermore, it is worth noting that the third improved strategy significantly reduced false positive rate of PSA compared to the first and second improved strategies, therefore, it would be suggested as a potentially recommended risk-adapted screening strategy to improve the effectiveness of current PSA screening.

In addition to the above important findings, several limitations also worth noting. First, there is no independent external population to verify the current results of this study, especially the PRS-specific cut-off values of PSA. These may limit the generalization of these results to other population. However, the results of 2000 iterations of bootstrap resampling analyses well confirm the internal stability of these cut-off values (Supplementary Table S8). Second, lowering the PSA cut-off level may lead to potential false positives and overdiagnosis. Although the recommended third improved strategy did not significantly cause excessive false positive rate, it also did not significantly improve the detection of high-grade PCa. However, previous study suggested that genetically adjusted PSA was more predictive of high-risk PCa compared to unadjusted PSA (AUC: 0.755 vs. 0.738), thus avoiding a large number of unnecessary biopsies (Kachuri et al., 2023). Future studies with more sophisticated design and large sample size are needed to support the incorporation of gene-adjusted PSA into PCa screening strategies, which could further improve screening effectiveness and increase the detection rate of high-risk PCa.

In conclusion, this study proposes an improved risk-adapted PSA screening integrating both PRS-specific and age-specific variation. This risk-adapted screening strategy would not only reduce potential missed diagnosis of PCa, but also ensures high specificity, and does not cause excessive false positive. However, the recommended improved screening strategy did not improve the detection rates of high-grade PCa. If available and feasible, a combination of the PRS-specific and age-specific PSA and other indicators would be explored to further improve the screening effectiveness in the future.

## Data Availability

The original contributions presented in the study are included in the article/Supplementary Material, further inquiries can be directed to the corresponding author.

## Abbreviations

NPV, negative prediction value; PCa, prostate cancer; PRS, polygenic risk scores; PSA, prostate-specific antigen; PPV, positive prediction value; tdAUCs, time-dependent area under curves; tdROCs, time-dependent receiver-operating-characteristic curves.
