# Supplementary material for: Preliminary effects of risk-adapted PSA screening for prostate cancer after integrating PRS-specific and age-specific variation
Source: Front Genet. 2024 Aug 1;15:1387588. doi: 10.3389/fgene.2024.1387588 (PMC11324495; doi:10.3389/fgene.2024.1387588)
Supplement: Supplementary file 1 [file Table1.DOCX]

**S1 Fig. Flowchart of participants’ selection.** PLCO, the Prostate, Lung, Colorectal, and Ovarian (PLCO) Cancer Screening Trial; GWAS, genome-wide association studies; BQ, baseline questionnaire; PCa, prostate cancer.

**S1 Table. Characteristics and sources of selected SNPs in this study**

**S2 Table. Distribution of selected genetic variants between cases and controls.**

**S3 Table. Associations between selected genetic variants and prostate cancer.** *, ORs were calculated with univariate logistics regression.

**S4 Table. Associations between per allele of genetic variants and prostate cancer after harmonizing the direction of association and imputing missing data of index SNP with common homozygotes.** *, beta was the log OR of per-allele index SNP with outcome from univariate logistics regression.

**S2 Fig. Accuracy of prostate cancer predicted by different polygenic risk scores (PRS).**

Unweighted PRS1 and PRS2 were calculated as the sum of number of risk allele from all selected SNPs and SNPs significantly associated with prostate cancer. Weighted PRS3 and PRS4 were calculated as the sum of risk allele from all selected SNPs and validated SNPs weighted with beta of SNP from univariate logistics regression.

**S5 Table. Baseline characteristics associated with the incidence of prostate cancer.** PC, prostate cancer; PY, person-year; IR, incidence rate; ^a^Missing data in the index variable were not shown; ^b^adjusted all index variables listed in the table, and missing data of each variable were coded as independent group in the multivariable COX regression.

**S6 Table. Accuracy of 10-year incidence risk of prostate cancer predicted by different polygenic risk scores (PRS).** AUCs were compared with DeLong test.

**S7 Table. Differences in incidence and mortality of prostate cancer in different genetic risk groups.**a, adjusted available variables associated with prostate cancer listed in S5 Table, and missing data of each variable were coded as independent group in the multivariable COX regression.

**S8 Table. Bootstrap resampling analyses on overall and age-specific cut-off values of PSA screening for PCa with 2000 iterations by genetic risks.** 95%CI, 95% confidential interval.


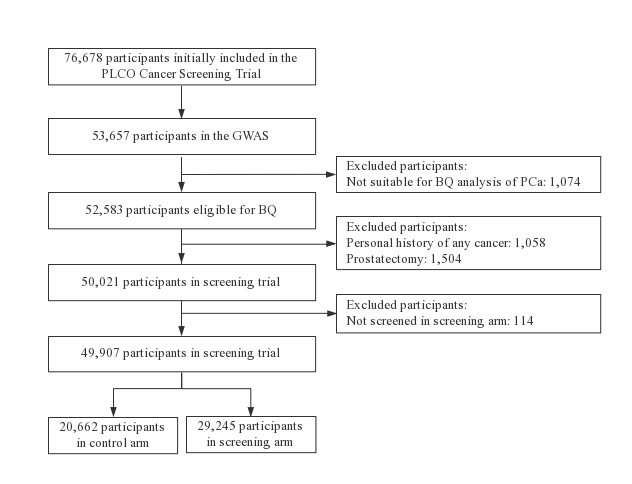


**S1 Fig. Flowchart of participants’ selection.**

PLCO, the Prostate, Lung, Colorectal, and Ovarian (PLCO) Cancer Screening Trial; GWAS, genome-wide association studies; BQ, baseline questionnaire; PCa, prostate cancer.

## S1Table. Characteristics and sources of selected SNPs in this study.

| rs number | Minor Allele | Major Allele | Nearby Gene | GWAS | | |
| --- | --- | --- | --- | --- | --- | --- |
|  |  |  |  | PMID | Year | Reference |
| rs4430796 | G | A | HNF1B | 17603485 | 2007 | [1] |
| rs4466137 | G | T | HAPLN1 | 17903305 | 2007 | [2] |
| rs10498792 | C | T | PKHD1 | 17903305 | 2007 |  |
| rs4962416 | C | T | CTBP2 | 18264096 | 2008 | [3] |
| rs10993994 | G | T | MSMB | 18264096 | 2008 |  |
| rs9364554 | T | C | SLC22A3 | 18264097 | 2008 | [4] |
| rs6465657 | C | T | LMTK2 | 18264097 | 2008 |  |
| rs1016343 | T | C | PRNCR1, PCAT1, CASC19 | 18264097 | 2008 |  |
| rs4242384 | C | A | CASC8 - CASC11 | 18264097 | 2008 |  |
| rs7501939 | C | T | HNF1B | 18264097 | 2008 |  |
| rs2735839 | G | A | - | 18264097 | 2008 |  |
| rs721048 | A | G | EHBP1 | 18264098 | 2008 | [5] |
| rs5945572 | A | G | EZHIP - NUDT11 | 18264098 | 2008 |  |
| rs9623117 | T | C | TNRC6B | 19117981 | 2009 | [6] |
| rs12621278 | A | G | ITGA6 | 19767753 | 2009 | [7] |
| rs17181170 | A | G | LINC00506 | 19767753 | 2009 |  |
| rs12500426 | C | A | PDLIM5 | 19767753 | 2009 |  |
| rs17021918 | C | T | PDLIM5 | 19767753 | 2009 |  |
| rs7679673 | C | A | RNU6-351P - TET2 | 19767753 | 2009 |  |
| rs12155172 | A | G | LINC01162 | 19767753 | 2009 |  |
| rs1512268 | T | C | SINHCAFP3 - NKX3-1 | 19767753 | 2009 |  |
| rs3123078 | T | C | RPL23AP61 - AGAP7P | 19767753 | 2009 |  |
| rs7130881 | G | A | SMIM38 - MYEOV | 19767753 | 2009 |  |
| rs5759167 | G | T | TTLL1 - BIK | 19767753 | 2009 |  |
| rs10934853 | A | C | EEFSEC | 19767754 | 2009 | [8] |
| rs16902094 | A | G | PCAT1, CASC8 | 19767754 | 2009 |  |
| rs445114 | T | C | CASC8, PCAT1 | 19767754 | 2009 |  |
| rs11228565 | A | G | SMIM38 - MYEOV | 19767754 | 2009 |  |
| rs8102476 | C | T | SPINT2 | 19767754 | 2009 |  |
| rs13385191 | A | G | LDAH | 20676098 | 2010 | [9] |
| rs9284813 | G | A | LINC00506 | 20676098 | 2010 |  |
| rs12653946 | T | C | CTD-2194D22.4 | 20676098 | 2010 |  |
| rs1983891 | C | T | FOXP4 | 20676098 | 2010 |  |
| rs339331 | T | C | RFX6 | 20676098 | 2010 |  |
| rs1456315 | T | C | PCAT1, CASC19, PRNCR1 | 20676098 | 2010 |  |
| rs9600079 | G | T | RNU6-66P - RNU4-10P | 20676098 | 2010 |  |
| rs7210100 | A | G | ZNF652 | 21602798 | 2011 | [10] |
| rs902774 | A | G | KRT78 - RPL7P41 | 21743057 | 2011 | [11] |
| rs2292884 | A | G | MLPH | 21743057 | 2011 |  |
| rs7584330 | G | A | COL6A3 - MLPH | 21743467 | 2011 |  |
| rs6763931 | A | G | ZBTB38 | 21743467 | 2011 |  |
| rs2121875 | C | A | FGF10 | 21743467 | 2011 |  |
| rs130067 | G | T | CCHCR1 | 21743467 | 2011 |  |
| rs10875943 | T | C | TUBA1C - TROAP-AS1 | 21743467 | 2011 |  |
| rs5919432 | T | C | BMI1P1 - NA | 21743467 | 2011 |  |
| rs78378222 | T | G | TP53 | 21946351 | 2011 | [12] |
| rs11199874 | G | A | LINC01153 - NA | 22130093 | 2011 | [13] |
| rs4775302 | A | G | MTND5P40 - NA | 22130093 | 2011 |  |
| rs17023900 | G | A | PPATP1 - LINC00506 | 22923026 | 2012 | [14] |
| rs10503733 | T | G | SINHCAFP3 - NKX3-1 | 22923026 | 2012 |  |
| rs10505483 | T | C | CASC19, PCAT1 | 22923026 | 2012 |  |
| rs817826 | T | C | RAD23B - LINC01509 | 23023329 | 2012 | [15] |
| rs103294 | T | C | MIR4752 - LILRA5 | 23023329 | 2012 |  |
| rs11672691 | G | A | PCAT19 | 23065704 | 2012 | [16] |
| rs188140481 | T | A | PCAT1, CASC19 | 23104005 | 2012 | [17] |
| rs4245739 | A | C | MDM4 | 23535732 | 2013 | [18] |
| rs11902236 | T | C | GRHL1 | 23535732 | 2013 |  |
| rs3771570 | C | T | FARP2 | 23535732 | 2013 |  |
| rs1894292 | G | A | AFM | 23535732 | 2013 |  |
| rs6869841 | C | T | NA - BOD1 | 23535732 | 2013 |  |
| rs3096702 | A | G | NOTCH4 - TSBP1-AS1 | 23535732 | 2013 |  |
| rs2273669 | G | A | ARMC2 | 23535732 | 2013 |  |
| rs1933488 | A | G | RGS17 | 23535732 | 2013 |  |
| rs11135910 | T | C | EBF2 | 23535732 | 2013 |  |
| rs3850699 | A | G | TRIM8 | 23535732 | 2013 |  |
| rs11568818 | C | T | MMP7 - MMP20 | 23535732 | 2013 |  |
| rs1270884 | G | A | LINC02459 - NA | 23535732 | 2013 |  |
| rs8008270 | C | T | FERMT2 | 23535732 | 2013 |  |
| rs7141529 | T | C | RAD51B | 23535732 | 2013 |  |
| rs684232 | C | T | VPS53 | 23535732 | 2013 |  |
| rs11650494 | A | G | FLJ40194 | 23535732 | 2013 |  |
| rs7241993 | C | T | SALL3 - ATP9B | 23535732 | 2013 |  |
| rs2427345 | C | T | RBBP8NL - GATA5 | 23535732 | 2013 |  |
| rs6062509 | T | G | ZGPAT | 23535732 | 2013 |  |
| rs2405942 | A | G | SHROOM2 | 23535732 | 2013 |  |
| rs7611694 | A | C | SIDT1 | 23535732 | 2013 |  |
| rs10069690 | C | T | TERT | 23535824 | 2013 | [19] |
| rs7126629 | A | C | MIR4686 - ASCL2 | 24740154 | 2014 | [20] |
| rs17632542 | T | C | KLK3 | 24740154 | 2014 |  |
| rs636291 | A | G | PEX14 | 25217961 | 2014 | [21] |
| rs17599629 | G | A | GOLPH3L | 25217961 | 2014 |  |
| rs9287719 | T | C | NOL10 | 25217961 | 2014 |  |
| rs10009409 | T | C | RNU4ATAC9P - RNU6ATAC5P | 25217961 | 2014 |  |
| rs17694493 | T | C | CDKN2B-AS1 | 25217961 | 2014 |  |
| rs76934034 | T | C | MARCHF8 | 25217961 | 2014 |  |
| rs11214775 | G | A | HTR3B | 25217961 | 2014 |  |
| rs7153648 | G | C | SIX1 | 25217961 | 2014 |  |
| rs8014671 | G | A | RN7SL77P - TTC9 | 25217961 | 2014 |  |
| rs1041449 | G | A | TMPRSS2 | 25217961 | 2014 |  |
| rs2807031 | T | C | XAGE3 | 25217961 | 2014 |  |
| rs6625711 |  | A | TEX11 - SLC7A3 | 25217961 | 2014 |  |
| rs1058205 | T | C | KLK3 | 25691096 | 2015 | [22] |
| rs71277158 | A | G | PRKCI | 25939597 | 2015 | [23] |
| rs78943174 | T | C | NAALADL2 | 25939597 | 2015 |  |
| rs7725218 | G | A | TERT | 25939597 | 2015 |  |
| rs35148638 | C | A | RASA1 | 25939597 | 2015 |  |
| rs7929962 | T | C | SMIM38 - MYEOV | 25939597 | 2015 |  |
| rs10774740 | G | T | GLULP5 - LINC02459 | 25939597 | 2015 |  |
| rs17765344 | A | G | CASC17 | 25939597 | 2015 |  |
| rs62113212 | C | T | KLK3 | 25939597 | 2015 |  |
| rs8064454 | C | A | HNF1B | 25939597 | 2015 |  |
| rs4646284 | D | I | SLC22A1 - SLC22A2 | 26034056 | 2015 | [24] |
| rs2659124 | T | A | KLK15 - KLK3 | 26034056 | 2015 |  |
| rs4749884 | C | A | LINC02663 | 26443449 | 2015 | [25] |
| rs10505477 | A | G | POU5F1B, PCAT1, CASC8 | 28139693 | 2017 | [26] |
| rs7758229 | G | T | SLC22A3 | 28827750 | 2017 | [27] |
| rs6431219 | T | C | BIN1 | 29356057 | 2018 | [28] |
| rs7258285 | G | A | ZIM2-AS1 | 29356057 | 2018 |  |
| rs11691517 | T | G | BCL2L11, MIR4435-2HG | 29892016 | 2018 | [29] |
| rs76551843 | A | G | DOCK2 | 29892016 | 2018 |  |
| rs4976790 | T | G | COL23A1 | 29892016 | 2018 |  |
| rs7767188 | G | A | TRIM31, TRIM31-AS1, | 29892016 | 2018 |  |
| rs12665339 | G | A | ATAT1 | 29892016 | 2018 |  |
| rs9296068 | T | G | HLA-DOA - HLA-DPA1 | 29892016 | 2018 |  |
| rs17621345 | A | C | SUGCT | 29892016 | 2018 |  |
| rs1935581 | C | T | RNLS | 29892016 | 2018 |  |
| rs61890184 | A | G | PPFIBP2 | 29892016 | 2018 |  |
| rs2066827 | G | T | CDKN1B | 29892016 | 2018 |  |
| rs33984059 | A | G | RFX7 | 29892016 | 2018 |  |
| rs138213197 | T | C | HOXB13 | 29892016 | 2018 |  |
| rs2680708 | A | G | TSPOAP1-AS1, RNF43 | 29892016 | 2018 |  |
| rs61088131 | T | C | POU2F2 - DEDD2 | 29892016 | 2018 |  |
| rs2788524 | G | A | ARHGAP6 | 29892016 | 2018 |  |
| rs4554825 | C | T | LINC00856 | 31562322 | 2019 | [30] |
| rs2252004 | C | A | LOC124902516 | 31562322 | 2019 |  |
| rs138708 | G | A | SUN2 | 31562322 | 2019 |  |
| rs6808932 | A | C | SIDT1 | 33290408 | 2020 | [31] |
| rs62516032 | T | C | PCAT1, CASC8, POU5F1B | 33290408 | 2020 |  |
| rs3217992 | C | T | CDKN2B-AS1, CDKN2B | 33290408 | 2020 |  |
| rs17522122 | C | T | AKAP6 | 33290408 | 2020 |  |
| rs7236466 | G | T | ZNF236 | 33290408 | 2020 |  |
| rs9306894 | A | G | GDF7 | 33398198 | 2021 | [32] |
| rs12621900 | C | T | MYOSLID - LINC01802 | 33398198 | 2021 |  |
| rs77559646 | G | A | ANO7 | 33398198 | 2021 |  |
| rs77482050 | A | G | ANO7 | 33398198 | 2021 |  |
| rs7618603 | A | C | NA - RPL24P7 | 33398198 | 2021 |  |
| rs71595003 | G | A | TERT | 33398198 | 2021 |  |
| rs6955627 | C | T | CDK6-AS1 - RN7SL7P | 33398198 | 2021 |  |
| rs2572375 | C | T | TDH, TDH | 33398198 | 2021 |  |
| rs13380763 | C | T | LINC02183 - NA | 33398198 | 2021 |  |
| rs2659051 | G | C | KLK15 - KLK3 | 33398198 | 2021 |  |
| rs61752561 | G | A | KLK3 | 33398198 | 2021 |  |
| rs16902147 | C | T | CASC8, POU5F1B, PCAT1 | 34012061 | 2021 | [33] |
| rs6457327 | A | C | RNU6-1133P - C6orf15 | 34290314 | 2021 |  |
| rs377763 | C | A | NOTCH4 - TSBP1-AS1 | 34290314 | 2021 |  |
| rs1635553 | G | A | COL2A1 | 34290314 | 2021 |  |
| rs12322335 | C | T | PRPH - TROAP | 34290314 | 2021 |  |
| rs12610088 | T | C | PCAT19 - PLEKHA3P1 | 34290314 | 2021 |  |
| rs73140002 | C | T | LOC101927770 | 34290314 | 2021 |  |
| rs699664 | C | T | GGCX | 34594039 | 2021 | [34] |
| rs2823739 | A | G | MIR99AHG | 34594039 | 2021 |  |
| rs6820205 | C | T | MTNR1A | 34981446 | 2022 | [35] |
| rs4710983 | T | C | CASC15 | 34981446 | 2022 |  |

## S2 Table. Distribution of selected genetic variants between cases and controls.

| SNP | Group | Total | |  | Common  homozygotes | |  | Heterozygote | |  | Rare  homozygotes | | P value | Heterozygote/  Rare homozygotes | | P value |
| --- | --- | --- | --- | --- | --- | --- | --- | --- | --- | --- | --- | --- | --- | --- | --- | --- |
|  |  | N | % |  | N | % |  | N | % |  | N | % |  | N | % |  |
| rs11902236 | Non-case | 43812 | 88.0% |  | 22620 | 88.3% |  | 17465 | 87.7% |  | 3727 | 87.4% | 0.085 | 21192 | 87.7% | 0.034 |
|  | Case | 5976 | 12.0% |  | 2998 | 11.7% |  | 2440 | 12.3% |  | 538 | 12.6% |  | 2978 | 12.3% |  |
| rs9306894 | Non-case | 40268 | 95.9% |  | 16733 | 95.6% |  | 18163 | 96.1% |  | 5372 | 95.9% | 0.067 | 23535 | 96.0% | 0.024 |
|  | Case | 1737 | 4.1% |  | 769 | 4.4% |  | 739 | 3.9% |  | 229 | 4.1% |  | 968 | 4.0% |  |
| rs13385191 | Non-case | 40267 | 95.9% |  | 23152 | 95.8% |  | 14467 | 96.0% |  | 2648 | 95.9% | 0.786 | 17115 | 96.0% | 0.513 |
|  | Case | 1731 | 4.1% |  | 1009 | 4.2% |  | 608 | 4.0% |  | 114 | 4.1% |  | 722 | 4.0% |  |
| rs721048 | Non-case | 37782 | 87.8% |  | 26082 | 88.2% |  | 10540 | 87.1% |  | 1160 | 86.0% | 0.001 | 11700 | 87.0% | <0.001 |
|  | Case | 5256 | 12.2% |  | 3503 | 11.8% |  | 1564 | 12.9% |  | 189 | 14.0% |  | 1753 | 13.0% |  |
| rs699664 | Non-case | 43853 | 88.0% |  | 19538 | 87.8% |  | 19239 | 88.2% |  | 5076 | 88.0% | 0.453 | 24315 | 88.2% | 0.222 |
|  | Case | 5977 | 12.0% |  | 2713 | 12.2% |  | 2575 | 11.8% |  | 689 | 12.0% |  | 3264 | 11.8% |  |
| rs11691517 | Non-case | 39871 | 95.9% |  | 22553 | 95.7% |  | 14884 | 96.0% |  | 2434 | 96.1% | 0.332 | 17318 | 96.0% | 0.144 |
|  | Case | 1719 | 4.1% |  | 1003 | 4.3% |  | 618 | 4.0% |  | 98 | 3.9% |  | 716 | 4.0% |  |
| rs6431219 | Non-case | 37982 | 87.7% |  | 13533 | 87.9% |  | 18187 | 87.4% |  | 6262 | 88.0% | 0.265 | 24449 | 87.6% | 0.350 |
|  | Case | 5335 | 12.3% |  | 1866 | 12.1% |  | 2617 | 12.6% |  | 852 | 12.0% |  | 3469 | 12.4% |  |
| rs12621278 | Non-case | 40455 | 95.9% |  | 35437 | 95.8% |  | 4819 | 96.5% |  | 199 | 95.2% | 0.060 | 5018 | 96.4% | 0.028 |
|  | Case | 1739 | 4.1% |  | 1554 | 4.2% |  | 175 | 3.5% |  | 10 | 4.8% |  | 185 | 3.6% |  |
| rs12621900 | Non-case | 43893 | 88.0% |  | 25249 | 88.2% |  | 15887 | 87.8% |  | 2757 | 88.3% | 0.460 | 18644 | 87.9% | 0.324 |
|  | Case | 5963 | 12.0% |  | 3390 | 11.8% |  | 2207 | 12.2% |  | 366 | 11.7% |  | 2573 | 12.1% |  |
| rs7584330 | Non-case | 43892 | 88.0% |  | 24782 | 88.3% |  | 16025 | 88.0% |  | 3085 | 85.9% | <0.001 | 19110 | 87.7% | 0.029 |
|  | Case | 5974 | 12.0% |  | 3284 | 11.7% |  | 2183 | 12.0% |  | 507 | 14.1% |  | 2690 | 12.3% |  |
| rs77559646 | Non-case | 40321 | 95.9% |  | 38691 | 95.9% |  | 1610 | 95.5% |  | 20 | 95.2% | 0.727 | 1630 | 95.5% | 0.425 |
|  | Case | 1739 | 4.1% |  | 1662 | 4.1% |  | 76 | 4.5% |  | 1 | 4.8% |  | 77 | 4.5% |  |
| rs7618603 | Non-case | 43835 | 88.0% |  | 29290 | 88.3% |  | 12955 | 87.7% |  | 1590 | 85.6% | 0.001 | 14545 | 87.5% | 0.011 |
|  | Case | 5970 | 12.0% |  | 3890 | 11.7% |  | 1813 | 12.3% |  | 267 | 14.4% |  | 2080 | 12.5% |  |
| rs17023900 | Non-case | 43758 | 88.2% |  | 37091 | 88.4% |  | 6352 | 87.0% |  | 315 | 84.0% | <0.001 | 6667 | 86.9% | <0.001 |
|  | Case | 5868 | 11.8% |  | 4861 | 11.6% |  | 947 | 13.0% |  | 60 | 16.0% |  | 1007 | 13.1% |  |
| rs71277158 | Non-case | 43100 | 87.9% |  | 30917 | 87.5% |  | 11046 | 88.6% |  | 1137 | 91.8% | <0.001 | 12183 | 88.9% | <0.001 |
|  | Case | 5959 | 12.1% |  | 4436 | 12.5% |  | 1422 | 11.4% |  | 101 | 8.2% |  | 1523 | 11.1% |  |
| rs78943174 | Non-case | 37975 | 87.7% |  | 36987 | 87.7% |  | 981 | 86.7% |  | 7 | 77.8% | 0.411 | 988 | 86.7% | 0.291 |
|  | Case | 5335 | 12.3% |  | 5183 | 12.3% |  | 150 | 13.3% |  | 2 | 22.2% |  | 152 | 13.3% |  |
| rs10009409 | Non-case | 43909 | 88.0% |  | 20675 | 88.4% |  | 18623 | 87.7% |  | 4611 | 87.5% | 0.021 | 23234 | 87.6% | 0.006 |
|  | Case | 5980 | 12.0% |  | 2702 | 11.6% |  | 2619 | 12.3% |  | 659 | 12.5% |  | 3278 | 12.4% |  |
| rs1894292 | Non-case | 43152 | 87.9% |  | 12334 | 87.5% |  | 21228 | 87.9% |  | 9590 | 88.3% | 0.123 | 30818 | 88.0% | 0.088 |
|  | Case | 5963 | 12.1% |  | 1768 | 12.5% |  | 2926 | 12.1% |  | 1269 | 11.7% |  | 4195 | 12.0% |  |
| rs12500426 | Non-case | 40468 | 95.9% |  | 12057 | 96.0% |  | 19963 | 95.8% |  | 8448 | 95.7% | 0.515 | 28411 | 95.8% | 0.285 |
|  | Case | 1738 | 4.1% |  | 497 | 4.0% |  | 865 | 4.2% |  | 376 | 4.3% |  | 1241 | 4.2% |  |
| rs17021918 | Non-case | 40469 | 95.9% |  | 17256 | 95.5% |  | 18293 | 96.0% |  | 4920 | 96.6% | 0.001 | 23213 | 96.2% | 0.001 |
|  | Case | 1738 | 4.1% |  | 810 | 4.5% |  | 755 | 4.0% |  | 173 | 3.4% |  | 928 | 3.8% |  |
| rs7679673 | Non-case | 43550 | 88.3% |  | 14132 | 86.6% |  | 21015 | 89.0% |  | 8403 | 89.4% | <0.001 | 29418 | 89.1% | <0.001 |
|  | Case | 5792 | 11.7% |  | 2194 | 13.4% |  | 2597 | 11.0% |  | 1001 | 10.6% |  | 3598 | 10.9% |  |
| rs6820205 | Non-case | 42680 | 87.8% |  | 36831 | 87.7% |  | 5604 | 88.3% |  | 245 | 89.4% | 0.307 | 5849 | 88.3% | 0.152 |
|  | Case | 5928 | 12.2% |  | 5156 | 12.3% |  | 743 | 11.7% |  | 29 | 10.6% |  | 772 | 11.7% |  |
| rs10069690 | Non-case | 43881 | 88.0% |  | 23806 | 87.4% |  | 16687 | 88.7% |  | 3388 | 89.0% | <0.001 | 20075 | 88.8% | <0.001 |
|  | Case | 5973 | 12.0% |  | 3436 | 12.6% |  | 2120 | 11.3% |  | 417 | 11.0% |  | 2537 | 11.2% |  |
| rs7725218 | Non-case | 42749 | 87.8% |  | 17867 | 86.7% |  | 19306 | 88.3% |  | 5576 | 89.9% | <0.001 | 24882 | 88.7% | <0.001 |
|  | Case | 5926 | 12.2% |  | 2742 | 13.3% |  | 2556 | 11.7% |  | 628 | 10.1% |  | 3184 | 11.3% |  |
| rs71595003 | Non-case | 40445 | 95.9% |  | 38451 | 95.9% |  | 1975 | 96.2% |  | 19 | 90.5% | 0.375 | 1994 | 96.1% | 0.607 |
|  | Case | 1739 | 4.1% |  | 1658 | 4.1% |  | 79 | 3.8% |  | 2 | 9.5% |  | 81 | 3.9% |  |
| rs4466137 | Non-case | 39099 | 95.9% |  | 22595 | 95.8% |  | 14193 | 96.1% |  | 2311 | 95.7% | 0.281 | 16504 | 96.0% | 0.185 |
|  | Case | 1683 | 4.1% |  | 1000 | 4.2% |  | 579 | 3.9% |  | 104 | 4.3% |  | 683 | 4.0% |  |
| rs35148638 | Non-case | 38715 | 87.9% |  | 21813 | 87.6% |  | 14409 | 88.4% |  | 2493 | 87.0% | 0.034 | 16902 | 88.2% | 0.093 |
|  | Case | 5346 | 12.1% |  | 3077 | 12.4% |  | 1898 | 11.6% |  | 371 | 13.0% |  | 2269 | 11.8% |  |
| rs76551843 | Non-case | 40466 | 95.9% |  | 39862 | 95.8% |  | 597 | 98.5% |  | 7 | 100.0% | 0.004 | 604 | 98.5% | 0.001 |
|  | Case | 1739 | 4.1% |  | 1730 | 4.2% |  | 9 | 1.5% |  | 0 | 0.0% |  | 9 | 1.5% |  |
| rs6869841 | Non-case | 40327 | 95.9% |  | 25207 | 96.1% |  | 13236 | 95.6% |  | 1884 | 94.4% | <0.001 | 15120 | 95.4% | <0.001 |
|  | Case | 1739 | 4.1% |  | 1012 | 3.9% |  | 616 | 4.4% |  | 111 | 5.6% |  | 727 | 4.6% |  |
| rs4976790 | Non-case | 43892 | 88.0% |  | 33382 | 88.1% |  | 9727 | 87.9% |  | 783 | 85.1% | 0.019 | 10510 | 87.7% | 0.186 |
|  | Case | 5982 | 12.0% |  | 4503 | 11.9% |  | 1342 | 12.1% |  | 137 | 14.9% |  | 1479 | 12.3% |  |
| rs4710983 | Non-case | 37738 | 87.6% |  | 24211 | 87.6% |  | 11534 | 87.6% |  | 1993 | 88.5% | 0.445 | 13527 | 87.7% | 0.675 |
|  | Case | 5322 | 12.4% |  | 3430 | 12.4% |  | 1633 | 12.4% |  | 259 | 11.5% |  | 1892 | 12.3% |  |
| rs7767188 | Non-case | 40424 | 95.9% |  | 26099 | 95.9% |  | 12756 | 95.8% |  | 1569 | 95.8% | 0.896 | 14325 | 95.8% | 0.642 |
|  | Case | 1737 | 4.1% |  | 1112 | 4.1% |  | 556 | 4.2% |  | 69 | 4.2% |  | 625 | 4.2% |  |
| rs12665339 | Non-case | 38700 | 87.8% |  | 27229 | 88.0% |  | 10393 | 87.4% |  | 1078 | 88.1% | 0.266 | 11471 | 87.5% | 0.137 |
|  | Case | 5357 | 12.2% |  | 3716 | 12.0% |  | 1495 | 12.6% |  | 146 | 11.9% |  | 1641 | 12.5% |  |
| rs6457327 | Non-case | 43892 | 88.0% |  | 17013 | 88.5% |  | 20436 | 87.7% |  | 6443 | 87.9% | 0.033 | 26879 | 87.7% | 0.010 |
|  | Case | 5981 | 12.0% |  | 2215 | 11.5% |  | 2876 | 12.3% |  | 890 | 12.1% |  | 3766 | 12.3% |  |
| rs130067 | Non-case | 38656 | 87.9% |  | 24616 | 87.9% |  | 12384 | 87.9% |  | 1656 | 87.6% | 0.916 | 14040 | 87.9% | 0.893 |
|  | Case | 5336 | 12.1% |  | 3403 | 12.1% |  | 1699 | 12.1% |  | 234 | 12.4% |  | 1933 | 12.1% |  |
| rs3096702 | Non-case | 37917 | 87.7% |  | 16242 | 87.9% |  | 16764 | 87.6% |  | 4911 | 87.4% | 0.514 | 21675 | 87.5% | 0.272 |
|  | Case | 5333 | 12.3% |  | 2242 | 12.1% |  | 2382 | 12.4% |  | 709 | 12.6% |  | 3091 | 12.5% |  |
| rs377763 | Non-case | 43874 | 88.0% |  | 27626 | 87.9% |  | 14323 | 88.2% |  | 1925 | 87.6% | 0.597 | 16248 | 88.1% | 0.522 |
|  | Case | 5977 | 12.0% |  | 3789 | 12.1% |  | 1916 | 11.8% |  | 272 | 12.4% |  | 2188 | 11.9% |  |
| rs9296068 | Non-case | 43916 | 88.0% |  | 18192 | 87.8% |  | 19788 | 88.0% |  | 5936 | 88.6% | 0.246 | 25724 | 88.1% | 0.301 |
|  | Case | 5982 | 12.0% |  | 2520 | 12.2% |  | 2698 | 12.0% |  | 764 | 11.4% |  | 3462 | 11.9% |  |
| rs10498792 | Non-case | 43858 | 88.1% |  | 34332 | 88.1% |  | 8908 | 87.8% |  | 618 | 88.4% | 0.653 | 9526 | 87.8% | 0.432 |
|  | Case | 5950 | 11.9% |  | 4631 | 11.9% |  | 1238 | 12.2% |  | 81 | 11.6% |  | 1319 | 12.2% |  |
| rs4646284 | Non-case | 40324 | 95.9% |  | 20413 | 96.1% |  | 16399 | 95.7% |  | 3512 | 95.3% | 0.034 | 19911 | 95.6% | 0.017 |
|  | Case | 1738 | 4.1% |  | 829 | 3.9% |  | 737 | 4.3% |  | 172 | 4.7% |  | 909 | 4.4% |  |
| rs17621345 | Non-case | 43895 | 88.0% |  | 25074 | 87.8% |  | 16103 | 88.3% |  | 2718 | 88.0% | 0.304 | 18821 | 88.3% | 0.139 |
|  | Case | 5969 | 12.0% |  | 3470 | 12.2% |  | 2130 | 11.7% |  | 369 | 12.0% |  | 2499 | 11.7% |  |
| rs6955627 | Non-case | 43885 | 88.0% |  | 35285 | 88.0% |  | 7836 | 88.1% |  | 764 | 87.4% | 0.811 | 8600 | 88.1% | 0.866 |
|  | Case | 5973 | 12.0% |  | 4808 | 12.0% |  | 1055 | 11.9% |  | 110 | 12.6% |  | 1165 | 11.9% |  |
| rs2572375 | Non-case | 40285 | 95.9% |  | 21453 | 95.4% |  | 15625 | 96.4% |  | 3207 | 96.7% | <0.001 | 18832 | 96.4% | <0.001 |
|  | Case | 1734 | 4.1% |  | 1037 | 4.6% |  | 587 | 3.6% |  | 110 | 3.3% |  | 697 | 3.6% |  |
| rs1016343 | Non-case | 43916 | 88.0% |  | 28242 | 89.0% |  | 13949 | 86.7% |  | 1725 | 83.6% | <0.001 | 15674 | 86.3% | <0.001 |
|  | Case | 5984 | 12.0% |  | 3502 | 11.0% |  | 2143 | 13.3% |  | 339 | 16.4% |  | 2482 | 13.7% |  |
| rs16902094 | Non-case | 40316 | 95.9% |  | 29859 | 96.0% |  | 9645 | 95.6% |  | 812 | 96.1% | 0.288 | 10457 | 95.6% | 0.156 |
|  | Case | 1737 | 4.1% |  | 1260 | 4.0% |  | 444 | 4.4% |  | 33 | 3.9% |  | 477 | 4.4% |  |
| rs445114 | Non-case | 43905 | 88.0% |  | 16636 | 87.1% |  | 20548 | 88.3% |  | 6721 | 89.4% | <0.001 | 27269 | 88.6% | <0.001 |
|  | Case | 5971 | 12.0% |  | 2456 | 12.9% |  | 2717 | 11.7% |  | 798 | 10.6% |  | 3515 | 11.4% |  |
| rs62516032 | Non-case | 43172 | 87.9% |  | 20633 | 87.2% |  | 18389 | 88.3% |  | 4150 | 89.2% | <0.001 | 22539 | 88.5% | <0.001 |
|  | Case | 5962 | 12.1% |  | 3022 | 12.8% |  | 2438 | 11.7% |  | 502 | 10.8% |  | 2940 | 11.5% |  |
| rs10505477 | Non-case | 43109 | 87.9% |  | 40462 | 88.1% |  | 2546 | 85.0% |  | 101 | 83.5% | <0.001 | 2647 | 84.9% | <0.001 |
|  | Case | 5934 | 12.1% |  | 5464 | 11.9% |  | 450 | 15.0% |  | 20 | 16.5% |  | 470 | 15.1% |  |
| rs4242384 | Non-case | 43909 | 88.0% |  | 10922 | 85.5% |  | 21711 | 88.3% |  | 11276 | 90.0% | <0.001 | 32987 | 88.9% | <0.001 |
|  | Case | 5984 | 12.0% |  | 1847 | 14.5% |  | 2880 | 11.7% |  | 1257 | 10.0% |  | 4137 | 11.1% |  |
| rs3217992 | Non-case | 43158 | 87.9% |  | 34980 | 88.7% |  | 7727 | 84.8% |  | 451 | 80.2% | <0.001 | 8178 | 84.5% | <0.001 |
|  | Case | 5936 | 12.1% |  | 4441 | 11.3% |  | 1384 | 15.2% |  | 111 | 19.8% |  | 1495 | 15.5% |  |
| rs17694493 | Non-case | 38557 | 87.8% |  | 15234 | 87.6% |  | 17754 | 87.9% |  | 5569 | 88.2% | 0.339 | 23323 | 88.0% | 0.178 |
|  | Case | 5342 | 12.2% |  | 2162 | 12.4% |  | 2436 | 12.1% |  | 744 | 11.8% |  | 3180 | 12.0% |  |
| rs817826 | Non-case | 40360 | 95.9% |  | 30459 | 95.9% |  | 9198 | 95.8% |  | 703 | 95.0% | 0.398 | 9901 | 95.7% | 0.386 |
|  | Case | 1733 | 4.1% |  | 1292 | 4.1% |  | 404 | 4.2% |  | 37 | 5.0% |  | 441 | 4.3% |  |
| rs4749884 | Non-case | 43918 | 88.0% |  | 32044 | 88.0% |  | 10892 | 88.2% |  | 982 | 86.8% | 0.353 | 11874 | 88.1% | 0.684 |
|  | Case | 5984 | 12.0% |  | 4381 | 12.0% |  | 1454 | 11.8% |  | 149 | 13.2% |  | 1603 | 11.9% |  |
| rs76934034 | Non-case | 43904 | 88.0% |  | 15872 | 87.5% |  | 20794 | 88.3% |  | 7238 | 88.3% | 0.020 | 28032 | 88.3% | 0.005 |
|  | Case | 5984 | 12.0% |  | 2274 | 12.5% |  | 2753 | 11.7% |  | 957 | 11.7% |  | 3710 | 11.7% |  |
| rs3123078 | Non-case | 40282 | 95.9% |  | 34172 | 95.7% |  | 5842 | 96.9% |  | 268 | 95.0% | <0.001 | 6110 | 96.9% | <0.001 |
|  | Case | 1728 | 4.1% |  | 1530 | 4.3% |  | 184 | 3.1% |  | 14 | 5.0% |  | 198 | 3.1% |  |
| rs4554825 | Non-case | 43914 | 88.0% |  | 14035 | 89.3% |  | 21484 | 88.1% |  | 8395 | 85.7% | <0.001 | 29879 | 87.4% | <0.001 |
|  | Case | 5977 | 12.0% |  | 1673 | 10.7% |  | 2908 | 11.9% |  | 1396 | 14.3% |  | 4304 | 12.6% |  |
| rs1935581 | Non-case | 43882 | 88.1% |  | 38186 | 88.0% |  | 5422 | 88.4% |  | 274 | 89.8% | 0.456 | 5696 | 88.4% | 0.322 |
|  | Case | 5942 | 11.9% |  | 5198 | 12.0% |  | 713 | 11.6% |  | 31 | 10.2% |  | 744 | 11.6% |  |
| rs3850699 | Non-case | 37539 | 88.0% |  | 13948 | 87.7% |  | 17544 | 88.2% |  | 6047 | 88.2% | 0.267 | 23591 | 88.2% | 0.105 |
|  | Case | 5122 | 12.0% |  | 1963 | 12.3% |  | 2347 | 11.8% |  | 812 | 11.8% |  | 3159 | 11.8% |  |
| rs2252004 | Non-case | 38617 | 87.9% |  | 19030 | 87.5% |  | 16136 | 88.0% |  | 3451 | 89.5% | 0.002 | 19587 | 88.2% | 0.019 |
|  | Case | 5332 | 12.1% |  | 2719 | 12.5% |  | 2207 | 12.0% |  | 406 | 10.5% |  | 2613 | 11.8% |  |
| rs61890184 | Non-case | 40467 | 95.9% |  | 31651 | 96.3% |  | 7931 | 95.2% |  | 885 | 89.0% | <0.001 | 8816 | 94.5% | <0.001 |
|  | Case | 1738 | 4.1% |  | 1229 | 3.7% |  | 400 | 4.8% |  | 109 | 11.0% |  | 509 | 5.5% |  |
| rs11214775 | Non-case | 43183 | 87.9% |  | 33210 | 88.1% |  | 9288 | 87.1% |  | 685 | 87.4% | 0.020 | 9973 | 87.1% | 0.005 |
|  | Case | 5963 | 12.1% |  | 4489 | 11.9% |  | 1375 | 12.9% |  | 99 | 12.6% |  | 1474 | 12.9% |  |
| rs2066827 | Non-case | 43903 | 88.0% |  | 21816 | 87.6% |  | 18243 | 88.2% |  | 3844 | 89.2% | 0.005 | 22087 | 88.4% | 0.006 |
|  | Case | 5974 | 12.0% |  | 3081 | 12.4% |  | 2429 | 11.8% |  | 464 | 10.8% |  | 2893 | 11.6% |  |
| rs1635553 | Non-case | 37512 | 87.7% |  | 21803 | 87.8% |  | 13081 | 87.5% |  | 2628 | 87.7% | 0.803 | 15709 | 87.6% | 0.538 |
|  | Case | 5276 | 12.3% |  | 3043 | 12.2% |  | 1864 | 12.5% |  | 369 | 12.3% |  | 2233 | 12.4% |  |
| rs10875943 | Non-case | 38708 | 87.8% |  | 11135 | 87.7% |  | 19176 | 87.9% |  | 8397 | 87.9% | 0.874 | 27573 | 87.9% | 0.620 |
|  | Case | 5355 | 12.2% |  | 1558 | 12.3% |  | 2636 | 12.1% |  | 1161 | 12.1% |  | 3797 | 12.1% |  |
| rs12322335 | Non-case | 41014 | 95.9% |  | 19750 | 96.5% |  | 16553 | 95.8% |  | 4711 | 93.7% | <0.001 | 21264 | 95.3% | <0.001 |
|  | Case | 1757 | 4.1% |  | 720 | 3.5% |  | 719 | 4.2% |  | 318 | 6.3% |  | 1037 | 4.7% |  |
| rs902774 | Non-case | 43900 | 88.0% |  | 29393 | 88.3% |  | 12904 | 87.5% |  | 1603 | 86.6% | 0.005 | 14507 | 87.4% | 0.002 |
|  | Case | 5975 | 12.0% |  | 3881 | 11.7% |  | 1846 | 12.5% |  | 248 | 13.4% |  | 2094 | 12.6% |  |
| rs10774740 | Non-case | 43883 | 88.0% |  | 32642 | 88.5% |  | 10348 | 86.6% |  | 893 | 86.2% | <0.001 | 11241 | 86.6% | <0.001 |
|  | Case | 5974 | 12.0% |  | 4231 | 11.5% |  | 1600 | 13.4% |  | 143 | 13.8% |  | 1743 | 13.4% |  |
| rs1270884 | Non-case | 43832 | 88.0% |  | 16221 | 87.2% |  | 20484 | 88.4% |  | 7127 | 88.9% | <0.001 | 27611 | 88.5% | <0.001 |
|  | Case | 5974 | 12.0% |  | 2388 | 12.8% |  | 2694 | 11.6% |  | 892 | 11.1% |  | 3586 | 11.5% |  |
| rs9600079 | Non-case | 40209 | 95.9% |  | 11416 | 95.1% |  | 19834 | 96.2% |  | 8959 | 96.1% | <0.001 | 28793 | 96.2% | <0.001 |
|  | Case | 1735 | 4.1% |  | 592 | 4.9% |  | 781 | 3.8% |  | 362 | 3.9% |  | 1143 | 3.8% |  |
| rs17522122 | Non-case | 43857 | 88.1% |  | 13221 | 88.0% |  | 21674 | 88.1% |  | 8962 | 88.3% | 0.843 | 30636 | 88.1% | 0.737 |
|  | Case | 5929 | 11.9% |  | 1800 | 12.0% |  | 2936 | 11.9% |  | 1193 | 11.7% |  | 4129 | 11.9% |  |
| rs8008270 | Non-case | 39681 | 95.9% |  | 11536 | 95.3% |  | 19665 | 96.0% |  | 8480 | 96.7% | <0.001 | 28145 | 96.2% | <0.001 |
|  | Case | 1690 | 4.1% |  | 569 | 4.7% |  | 829 | 4.0% |  | 292 | 3.3% |  | 1121 | 3.8% |  |
| rs7153648 | Non-case | 43907 | 88.0% |  | 29139 | 87.9% |  | 13127 | 88.2% |  | 1641 | 88.5% | 0.466 | 14768 | 88.3% | 0.230 |
|  | Case | 5984 | 12.0% |  | 4018 | 12.1% |  | 1752 | 11.8% |  | 214 | 11.5% |  | 1966 | 11.7% |  |
| rs7141529 | Non-case | 40322 | 95.9% |  | 33163 | 96.2% |  | 6679 | 94.5% |  | 480 | 89.4% | <0.001 | 7159 | 94.2% | <0.001 |
|  | Case | 1739 | 4.1% |  | 1296 | 3.8% |  | 386 | 5.5% |  | 57 | 10.6% |  | 443 | 5.8% |  |
| rs8014671 | Non-case | 43877 | 88.0% |  | 11694 | 87.8% |  | 21729 | 88.2% |  | 10454 | 88.0% | 0.521 | 32183 | 88.1% | 0.293 |
|  | Case | 5972 | 12.0% |  | 1630 | 12.2% |  | 2917 | 11.8% |  | 1425 | 12.0% |  | 4342 | 11.9% |  |
| rs4775302 | Non-case | 43880 | 88.0% |  | 14359 | 87.7% |  | 21242 | 88.1% |  | 8279 | 88.5% | 0.115 | 29521 | 88.2% | 0.066 |
|  | Case | 5959 | 12.0% |  | 2021 | 12.3% |  | 2863 | 11.9% |  | 1075 | 11.5% |  | 3938 | 11.8% |  |
| rs33984059 | Non-case | 40044 | 95.9% |  | 11471 | 96.1% |  | 19466 | 95.9% |  | 9107 | 95.6% | 0.173 | 28573 | 95.8% | 0.119 |
|  | Case | 1724 | 4.1% |  | 464 | 3.9% |  | 841 | 4.1% |  | 419 | 4.4% |  | 1260 | 4.2% |  |
| rs13380763 | Non-case | 40290 | 95.9% |  | 38554 | 95.8% |  | 1712 | 97.0% |  | 24 | 100.0% | 0.030 | 1736 | 97.0% | 0.011 |
|  | Case | 1739 | 4.1% |  | 1686 | 4.2% |  | 53 | 3.0% |  | 0 | 0.0% |  | 53 | 3.0% |  |
| rs684232 | Non-case | 43104 | 87.9% |  | 28892 | 87.7% |  | 12741 | 88.1% |  | 1471 | 88.6% | 0.339 | 14212 | 88.2% | 0.180 |
|  | Case | 5950 | 12.1% |  | 4040 | 12.3% |  | 1721 | 11.9% |  | 189 | 11.4% |  | 1910 | 11.8% |  |
| rs78378222 | Non-case | 43898 | 88.1% |  | 17236 | 88.6% |  | 20415 | 88.2% |  | 6247 | 86.2% | <0.001 | 26662 | 87.7% | 0.005 |
|  | Case | 5957 | 11.9% |  | 2226 | 11.4% |  | 2735 | 11.8% |  | 996 | 13.8% |  | 3731 | 12.3% |  |
| rs7501939 | Non-case | 40303 | 95.9% |  | 39457 | 95.9% |  | 839 | 93.7% |  | 7 | 100.0% | 0.004 | 846 | 93.8% | 0.001 |
|  | Case | 1729 | 4.1% |  | 1673 | 4.1% |  | 56 | 6.3% |  | 0 | 0.0% |  | 56 | 6.2% |  |
| rs138213197 | Non-case | 43847 | 88.2% |  | 15591 | 87.2% |  | 21139 | 88.5% |  | 7117 | 89.3% | <0.001 | 28256 | 88.7% | <0.001 |
|  | Case | 5889 | 11.8% |  | 2297 | 12.8% |  | 2743 | 11.5% |  | 849 | 10.7% |  | 3592 | 11.3% |  |
| rs11650494 | Non-case | 40288 | 95.9% |  | 40174 | 95.9% |  | 114 | 84.4% |  |  |  | <0.001 | 114 | 84.4% | <0.001 |
|  | Case | 1739 | 4.1% |  | 1718 | 4.1% |  | 21 | 15.6% |  |  |  |  | 21 | 15.6% |  |
| rs7210100 | Non-case | 43179 | 87.9% |  | 36497 | 88.1% |  | 6353 | 86.3% |  | 329 | 88.4% | <0.001 | 6682 | 86.4% | <0.001 |
|  | Case | 5967 | 12.1% |  | 4919 | 11.9% |  | 1005 | 13.7% |  | 43 | 11.6% |  | 1048 | 13.6% |  |
| rs2680708 | Non-case | 43882 | 88.0% |  | 43720 | 88.0% |  | 160 | 81.2% |  | 2 | 50.0% | 0.001 | 162 | 80.6% | 0.001 |
|  | Case | 5975 | 12.0% |  | 5936 | 12.0% |  | 37 | 18.8% |  | 2 | 50.0% |  | 39 | 19.4% |  |
| rs17765344 | Non-case | 43071 | 87.9% |  | 15404 | 87.8% |  | 20552 | 88.0% |  | 7115 | 87.7% | 0.710 | 27667 | 87.9% | 0.661 |
|  | Case | 5952 | 12.1% |  | 2146 | 12.2% |  | 2807 | 12.0% |  | 999 | 12.3% |  | 3806 | 12.1% |  |
| rs7236466 | Non-case | 43878 | 88.1% |  | 12663 | 88.9% |  | 21520 | 88.5% |  | 9695 | 86.3% | <0.001 | 31215 | 87.8% | 0.001 |
|  | Case | 5916 | 11.9% |  | 1585 | 11.1% |  | 2789 | 11.5% |  | 1542 | 13.7% |  | 4331 | 12.2% |  |
| rs7241993 | Non-case | 43894 | 88.0% |  | 15836 | 88.1% |  | 20906 | 88.0% |  | 7152 | 87.8% | 0.668 | 28058 | 87.9% | 0.467 |
|  | Case | 5981 | 12.0% |  | 2129 | 11.9% |  | 2855 | 12.0% |  | 997 | 12.2% |  | 3852 | 12.1% |  |
| rs12610088 | Non-case | 43870 | 88.1% |  | 20915 | 87.9% |  | 18494 | 88.2% |  | 4461 | 88.1% | 0.619 | 22955 | 88.2% | 0.335 |
|  | Case | 5937 | 11.9% |  | 2870 | 12.1% |  | 2467 | 11.8% |  | 600 | 11.9% |  | 3067 | 11.8% |  |
| rs61088131 | Non-case | 43464 | 88.1% |  | 16366 | 88.0% |  | 20325 | 88.3% |  | 6773 | 87.8% | 0.342 | 27098 | 88.2% | 0.439 |
|  | Case | 5865 | 11.9% |  | 2239 | 12.0% |  | 2685 | 11.7% |  | 941 | 12.2% |  | 3626 | 11.8% |  |
| rs2659051 | Non-case | 39731 | 95.9% |  | 26631 | 96.3% |  | 11156 | 95.5% |  | 1944 | 91.6% | <0.001 | 13100 | 94.9% | <0.001 |
|  | Case | 1720 | 4.1% |  | 1016 | 3.7% |  | 526 | 4.5% |  | 178 | 8.4% |  | 704 | 5.1% |  |
| rs2659124 | Non-case | 40449 | 95.9% |  | 24797 | 95.7% |  | 13611 | 96.0% |  | 2041 | 96.9% | 0.017 | 15652 | 96.1% | 0.036 |
|  | Case | 1738 | 4.1% |  | 1109 | 4.3% |  | 564 | 4.0% |  | 65 | 3.1% |  | 629 | 3.9% |  |
| rs62113212 | Non-case | 40150 | 95.9% |  | 28659 | 95.8% |  | 10335 | 95.8% |  | 1156 | 97.1% | 0.076 | 11491 | 95.9% | 0.694 |
|  | Case | 1735 | 4.1% |  | 1246 | 4.2% |  | 455 | 4.2% |  | 34 | 2.9% |  | 489 | 4.1% |  |
| rs61752561 | Non-case | 40326 | 95.9% |  | 34616 | 95.7% |  | 5443 | 97.0% |  | 267 | 98.9% | <0.001 | 5710 | 97.1% | <0.001 |
|  | Case | 1739 | 4.1% |  | 1569 | 4.3% |  | 167 | 3.0% |  | 3 | 1.1% |  | 170 | 2.9% |  |
| rs17632542 | Non-case | 43183 | 87.9% |  | 39972 | 87.9% |  | 3136 | 88.0% |  | 75 | 91.5% | 0.590 | 3211 | 88.1% | 0.700 |
|  | Case | 5962 | 12.1% |  | 5527 | 12.1% |  | 428 | 12.0% |  | 7 | 8.5% |  | 435 | 11.9% |  |
| rs103294 | Non-case | 41168 | 95.9% |  | 35373 | 95.7% |  | 5527 | 97.0% |  | 268 | 98.9% | <0.001 | 5795 | 97.1% | <0.001 |
|  | Case | 1754 | 4.1% |  | 1581 | 4.3% |  | 170 | 3.0% |  | 3 | 1.1% |  | 173 | 2.9% |  |
| rs7258285 | Non-case | 38704 | 87.8% |  | 23914 | 87.7% |  | 12572 | 88.0% |  | 2218 | 88.6% | 0.297 | 14790 | 88.1% | 0.174 |
|  | Case | 5353 | 12.2% |  | 3359 | 12.3% |  | 1708 | 12.0% |  | 286 | 11.4% |  | 1994 | 11.9% |  |
| rs73140002 | Non-case | 43886 | 88.0% |  | 17443 | 87.9% |  | 20258 | 88.1% |  | 6185 | 88.0% | 0.933 | 26443 | 88.1% | 0.722 |
|  | Case | 5977 | 12.0% |  | 2390 | 12.1% |  | 2745 | 11.9% |  | 842 | 12.0% |  | 3587 | 11.9% |  |
| rs2427345 | Non-case | 37954 | 87.7% |  | 35223 | 87.7% |  | 2650 | 87.0% |  | 81 | 87.1% | 0.450 | 2731 | 87.0% | 0.206 |
|  | Case | 5329 | 12.3% |  | 4920 | 12.3% |  | 397 | 13.0% |  | 12 | 12.9% |  | 409 | 13.0% |  |
| rs6062509 | Non-case | 43908 | 88.0% |  | 17487 | 88.1% |  | 20193 | 88.1% |  | 6228 | 87.6% | 0.499 | 26421 | 88.0% | 0.541 |
|  | Case | 5970 | 12.0% |  | 2353 | 11.9% |  | 2737 | 11.9% |  | 880 | 12.4% |  | 3617 | 12.0% |  |
| rs2823739 | Non-case | 40467 | 95.9% |  | 19560 | 95.4% |  | 16798 | 96.3% |  | 4109 | 96.7% | <0.001 | 20907 | 96.3% | <0.001 |
|  | Case | 1739 | 4.1% |  | 944 | 4.6% |  | 653 | 3.7% |  | 142 | 3.3% |  | 795 | 3.7% |  |
| rs1041449 | Non-case | 43880 | 88.0% |  | 37749 | 88.0% |  | 5863 | 88.0% |  | 268 | 88.7% | 0.927 | 6131 | 88.0% | 0.949 |
|  | Case | 5975 | 12.0% |  | 5142 | 12.0% |  | 799 | 12.0% |  | 34 | 11.3% |  | 833 | 12.0% |  |
| rs138708 | Non-case | 37793 | 87.7% |  | 12675 | 88.4% |  | 18270 | 87.5% |  | 6848 | 86.7% | 0.001 | 25118 | 87.3% | 0.001 |
|  | Case | 5322 | 12.3% |  | 1664 | 11.6% |  | 2606 | 12.5% |  | 1052 | 13.3% |  | 3658 | 12.7% |  |
| rs9623117 | Non-case | 42708 | 87.8% |  | 40755 | 87.7% |  | 1910 | 89.4% |  | 43 | 86.0% | 0.067 | 1953 | 89.3% | 0.027 |
|  | Case | 5940 | 12.2% |  | 5706 | 12.3% |  | 227 | 10.6% |  | 7 | 14.0% |  | 234 | 10.7% |  |
| rs5759167 | Non-case | 40454 | 95.9% |  | 24020 | 96.2% |  | 13728 | 96.1% |  | 2706 | 91.9% | <0.001 | 16434 | 95.4% | <0.001 |
|  | Case | 1739 | 4.1% |  | 950 | 3.8% |  | 550 | 3.9% |  | 239 | 8.1% |  | 789 | 4.6% |  |
| rs2405942 | Non-case | 43896 | 88.0% |  | 11587 | 86.5% |  | 21723 | 88.1% |  | 10586 | 89.5% | <0.001 | 32309 | 88.5% | <0.001 |
|  | Case | 5981 | 12.0% |  | 1803 | 13.5% |  | 2934 | 11.9% |  | 1244 | 10.5% |  | 4178 | 11.5% |  |
| rs2788524 | Non-case | 43895 | 88.0% |  | 34707 | 88.0% |  | 6 | 100.0% |  | 9182 | 88.0% | 0.665 | 9188 | 88.0% | 0.980 |
|  | Case | 5971 | 12.0% |  | 4722 | 12.0% |  | 0 | 0.0% |  | 1249 | 12.0% |  | 1249 | 12.0% |  |
| rs5945572 | Non-case | 40651 | 96.0% |  | 34582 | 95.9% |  | 13 | 92.9% |  | 6056 | 96.6% | 0.031 | 6069 | 96.5% | 0.011 |
|  | Case | 1709 | 4.0% |  | 1492 | 4.1% |  | 1 | 7.1% |  | 216 | 3.4% |  | 217 | 3.5% |  |
| rs2807031 | Non-case | 38681 | 87.8% |  | 25633 | 88.5% |  | 5 | 100.0% |  | 13043 | 86.6% | <0.001 | 13048 | 86.6% | <0.001 |
|  | Case | 5350 | 12.2% |  | 3334 | 11.5% |  | 0 | 0.0% |  | 2016 | 13.4% |  | 2016 | 13.4% |  |
| rs5919432 | Non-case | 40432 | 95.9% |  | 33316 | 95.9% |  | 4 | 100.0% |  | 7112 | 95.7% | 0.651 | 7116 | 95.7% | 0.413 |
|  | Case | 1737 | 4.1% |  | 1418 | 4.1% |  | 0 | 0.0% |  | 319 | 4.3% |  | 319 | 4.3% |  |
| rs6625711 | Non-case | 43021 | 87.8% |  | 33951 | 87.7% |  | 5 | 100.0% |  | 9065 | 88.1% | 0.415 | 9070 | 88.1% | 0.295 |
|  | Case | 5963 | 12.2% |  | 4741 | 12.3% |  | 0 | 0.0% |  | 1222 | 11.9% |  | 1222 | 11.9% |  |
| rs10503733 | Non-case | 40289 | 95.9% |  | 40289 | 95.9% |  |  |  |  |  |  |  |  |  |  |
|  | Case | 1733 | 4.1% |  | 1733 | 4.1% |  |  |  |  |  |  |  |  |  |  |
| rs636291 | Non-case | 38712 | 87.8% |  | 22040 | 88.2% |  | 14318 | 87.5% |  | 2354 | 87.0% | 0.026 | 16672 | 87.4% | 0.009 |
|  | Case | 5355 | 12.2% |  | 2948 | 11.8% |  | 2054 | 12.5% |  | 353 | 13.0% |  | 2407 | 12.6% |  |
| rs17599629 | Non-case | 41176 | 95.9% |  | 18503 | 96.3% |  | 17867 | 95.9% |  | 4806 | 94.2% | <0.001 | 22673 | 95.6% | <0.001 |
|  | Case | 1756 | 4.1% |  | 701 | 3.7% |  | 758 | 4.1% |  | 297 | 5.8% |  | 1055 | 4.4% |  |
| rs4245739 | Non-case | 43762 | 88.0% |  | 27544 | 88.4% |  | 14290 | 87.3% |  | 1928 | 86.5% | <0.001 | 16218 | 87.2% | <0.001 |
|  | Case | 5976 | 12.0% |  | 3603 | 11.6% |  | 2071 | 12.7% |  | 302 | 13.5% |  | 2373 | 12.8% |  |
| rs9287719 | Non-case | 43900 | 88.0% |  | 24093 | 87.6% |  | 16719 | 88.6% |  | 3088 | 88.2% | 0.007 | 19807 | 88.5% | 0.002 |
|  | Case | 5977 | 12.0% |  | 3406 | 12.4% |  | 2157 | 11.4% |  | 414 | 11.8% |  | 2571 | 11.5% |  |
| rs77482050 | Non-case | 39727 | 95.9% |  | 11447 | 95.9% |  | 19719 | 95.8% |  | 8561 | 96.0% | 0.865 | 28280 | 95.9% | 0.994 |
|  | Case | 1707 | 4.1% |  | 492 | 4.1% |  | 856 | 4.2% |  | 359 | 4.0% |  | 1215 | 4.1% |  |
| rs9284813 | Non-case | 43183 | 87.9% |  | 42529 | 87.8% |  | 651 | 89.1% |  | 3 | 100.0% | 0.497 | 654 | 89.1% | 0.303 |
|  | Case | 5962 | 12.1% |  | 5882 | 12.2% |  | 80 | 10.9% |  | 0 | 0.0% |  | 80 | 10.9% |  |
| rs17181170 | Non-case | 43169 | 87.9% |  | 31548 | 88.1% |  | 10531 | 87.4% |  | 1090 | 84.8% | <0.001 | 11621 | 87.2% | 0.005 |
|  | Case | 5958 | 12.1% |  | 4251 | 11.9% |  | 1512 | 12.6% |  | 195 | 15.2% |  | 1707 | 12.8% |  |
| rs6808932 | Non-case | 43912 | 88.0% |  | 12198 | 87.2% |  | 21550 | 87.9% |  | 10164 | 89.2% | <0.001 | 31714 | 88.3% | <0.001 |
|  | Case | 5983 | 12.0% |  | 1791 | 12.8% |  | 2955 | 12.1% |  | 1237 | 10.8% |  | 4192 | 11.7% |  |
| rs10934853 | Non-case | 43904 | 88.0% |  | 15436 | 87.5% |  | 21006 | 88.1% |  | 7462 | 88.8% | 0.007 | 28468 | 88.3% | 0.009 |
|  | Case | 5974 | 12.0% |  | 2203 | 12.5% |  | 2833 | 11.9% |  | 938 | 11.2% |  | 3771 | 11.7% |  |
| rs6763931 | Non-case | 43888 | 88.0% |  | 22001 | 88.6% |  | 17550 | 87.6% |  | 4337 | 87.0% | <0.001 | 21887 | 87.5% | <0.001 |
|  | Case | 5981 | 12.0% |  | 2842 | 11.4% |  | 2492 | 12.4% |  | 647 | 13.0% |  | 3139 | 12.5% |  |
| rs12653946 | Non-case | 43912 | 88.0% |  | 13893 | 88.5% |  | 21111 | 88.0% |  | 8908 | 87.2% | 0.004 | 30019 | 87.8% | 0.015 |
|  | Case | 5981 | 12.0% |  | 1799 | 11.5% |  | 2870 | 12.0% |  | 1312 | 12.8% |  | 4182 | 12.2% |  |
| rs2121875 | Non-case | 43903 | 88.0% |  | 14867 | 89.1% |  | 21163 | 87.7% |  | 7873 | 87.0% | <0.001 | 29036 | 87.5% | <0.001 |
|  | Case | 5977 | 12.0% |  | 1826 | 10.9% |  | 2974 | 12.3% |  | 1177 | 13.0% |  | 4151 | 12.5% |  |
| rs1983891 | Non-case | 43883 | 88.1% |  | 18983 | 88.5% |  | 19375 | 88.0% |  | 5525 | 86.6% | <0.001 | 24900 | 87.7% | 0.008 |
|  | Case | 5954 | 11.9% |  | 2468 | 11.5% |  | 2632 | 12.0% |  | 854 | 13.4% |  | 3486 | 12.3% |  |
| rs2273669 | Non-case | 40467 | 95.9% |  | 21168 | 96.4% |  | 16103 | 95.6% |  | 3196 | 94.0% | <0.001 | 19299 | 95.3% | <0.001 |
|  | Case | 1739 | 4.1% |  | 787 | 3.6% |  | 748 | 4.4% |  | 204 | 6.0% |  | 952 | 4.7% |  |
| rs339331 | Non-case | 43159 | 88.1% |  | 31330 | 88.3% |  | 10830 | 87.7% |  | 999 | 86.0% | 0.029 | 11829 | 87.6% | 0.041 |
|  | Case | 5837 | 11.9% |  | 4163 | 11.7% |  | 1512 | 12.3% |  | 162 | 14.0% |  | 1674 | 12.4% |  |
| rs1933488 | Non-case | 43866 | 88.1% |  | 20977 | 87.6% |  | 18637 | 88.5% |  | 4252 | 89.6% | <0.001 | 22889 | 88.7% | <0.001 |
|  | Case | 5900 | 11.9% |  | 2982 | 12.4% |  | 2425 | 11.5% |  | 493 | 10.4% |  | 2918 | 11.3% |  |
| rs9364554 | Non-case | 43913 | 88.0% |  | 14121 | 87.4% |  | 21114 | 88.0% |  | 8678 | 89.0% | 0.001 | 29792 | 88.3% | 0.006 |
|  | Case | 5978 | 12.0% |  | 2028 | 12.6% |  | 2874 | 12.0% |  | 1076 | 11.0% |  | 3950 | 11.7% |  |
| rs12155172 | Non-case | 43869 | 88.1% |  | 23500 | 88.7% |  | 17040 | 87.6% |  | 3329 | 86.2% | <0.001 | 20369 | 87.3% | <0.001 |
|  | Case | 5939 | 11.9% |  | 2989 | 11.3% |  | 2417 | 12.4% |  | 533 | 13.8% |  | 2950 | 12.7% |  |
| rs6465657 | Non-case | 43906 | 88.1% |  | 26881 | 88.4% |  | 14930 | 87.6% |  | 2095 | 86.7% | 0.005 | 17025 | 87.5% | 0.003 |
|  | Case | 5953 | 11.9% |  | 3525 | 11.6% |  | 2106 | 12.4% |  | 322 | 13.3% |  | 2428 | 12.5% |  |
| rs1512268 | Non-case | 43872 | 88.0% |  | 11982 | 88.8% |  | 20643 | 87.9% |  | 11247 | 87.4% | 0.002 | 31890 | 87.7% | 0.002 |
|  | Case | 5972 | 12.0% |  | 1516 | 11.2% |  | 2832 | 12.1% |  | 1624 | 12.6% |  | 4456 | 12.3% |  |
| rs11135910 | Non-case | 38699 | 87.9% |  | 12740 | 89.2% |  | 18837 | 87.6% |  | 7122 | 86.4% | <0.001 | 25959 | 87.3% | <0.001 |
|  | Case | 5325 | 12.1% |  | 1542 | 10.8% |  | 2666 | 12.4% |  | 1117 | 13.6% |  | 3783 | 12.7% |  |
| rs1456315 | Non-case | 43911 | 88.0% |  | 31751 | 88.3% |  | 11109 | 87.1% |  | 1051 | 87.6% | 0.002 | 12160 | 87.2% | <0.001 |
|  | Case | 5983 | 12.0% |  | 4194 | 11.7% |  | 1640 | 12.9% |  | 149 | 12.4% |  | 1789 | 12.8% |  |
| rs10505483 | Non-case | 43832 | 88.1% |  | 39804 | 88.5% |  | 3678 | 84.5% |  | 350 | 78.5% | <0.001 | 4028 | 83.9% | <0.001 |
|  | Case | 5936 | 11.9% |  | 5163 | 11.5% |  | 677 | 15.5% |  | 96 | 21.5% |  | 773 | 16.1% |  |
| rs188140481 | Non-case | 43914 | 88.0% |  | 39926 | 88.4% |  | 3670 | 84.4% |  | 318 | 79.7% | <0.001 | 3988 | 84.0% | <0.001 |
|  | Case | 5981 | 12.0% |  | 5224 | 11.6% |  | 676 | 15.6% |  | 81 | 20.3% |  | 757 | 16.0% |  |
| rs11199874 | Non-case | 40260 | 95.9% |  | 39971 | 95.9% |  | 289 | 92.6% |  | 0 | 0.0% | <0.001 | 289 | 92.0% | 0.001 |
|  | Case | 1730 | 4.1% |  | 1705 | 4.1% |  | 23 | 7.4% |  | 2 | 100.0% |  | 25 | 8.0% |  |
| rs4962416 | Non-case | 40315 | 95.9% |  | 23062 | 95.7% |  | 14803 | 96.1% |  | 2450 | 96.4% | 0.034 | 17253 | 96.1% | 0.012 |
|  | Case | 1739 | 4.1% |  | 1048 | 4.3% |  | 599 | 3.9% |  | 92 | 3.6% |  | 691 | 3.9% |  |
| rs7126629 | Non-case | 43822 | 88.0% |  | 23893 | 88.5% |  | 16725 | 87.5% |  | 3204 | 87.3% | 0.001 | 19929 | 87.4% | <0.001 |
|  | Case | 5953 | 12.0% |  | 3091 | 11.5% |  | 2394 | 12.5% |  | 468 | 12.7% |  | 2862 | 12.6% |  |
| rs11228565 | Non-case | 40323 | 95.9% |  | 26514 | 96.3% |  | 12209 | 95.3% |  | 1600 | 94.2% | <0.001 | 13809 | 95.1% | <0.001 |
|  | Case | 1732 | 4.1% |  | 1027 | 3.7% |  | 607 | 4.7% |  | 98 | 5.8% |  | 705 | 4.9% |  |
| rs7929962 | Non-case | 43909 | 88.0% |  | 28641 | 88.6% |  | 13571 | 87.1% |  | 1697 | 85.4% | <0.001 | 15268 | 86.9% | <0.001 |
|  | Case | 5985 | 12.0% |  | 3689 | 11.4% |  | 2006 | 12.9% |  | 290 | 14.6% |  | 2296 | 13.1% |  |
| rs7130881 | Non-case | 43910 | 88.0% |  | 11937 | 89.8% |  | 21186 | 88.0% |  | 10787 | 86.1% | <0.001 | 31973 | 87.3% | <0.001 |
|  | Case | 5982 | 12.0% |  | 1350 | 10.2% |  | 2892 | 12.0% |  | 1740 | 13.9% |  | 4632 | 12.7% |  |
| rs11568818 | Non-case | 43899 | 88.0% |  | 31446 | 88.8% |  | 11356 | 86.3% |  | 1097 | 84.3% | <0.001 | 12453 | 86.1% | <0.001 |
|  | Case | 5959 | 12.0% |  | 3954 | 11.2% |  | 1801 | 13.7% |  | 204 | 15.7% |  | 2005 | 13.9% |  |
| rs8102476 | Non-case | 38678 | 87.9% |  | 12736 | 87.9% |  | 18360 | 87.6% |  | 7582 | 88.6% | 0.041 | 25942 | 87.9% | 0.853 |
|  | Case | 5320 | 12.1% |  | 1745 | 12.1% |  | 2603 | 12.4% |  | 972 | 11.4% |  | 3575 | 12.1% |  |
| rs11672691 | Non-case | 38708 | 87.8% |  | 11746 | 87.0% |  | 19022 | 88.0% |  | 7940 | 88.8% | <0.001 | 26962 | 88.2% | <0.001 |
|  | Case | 5355 | 12.2% |  | 1758 | 13.0% |  | 2597 | 12.0% |  | 1000 | 11.2% |  | 3597 | 11.8% |  |
| rs1058205 | Non-case | 43681 | 88.0% |  | 22823 | 87.6% |  | 16887 | 88.6% |  | 3971 | 87.6% | 0.005 | 20858 | 88.4% | 0.008 |
|  | Case | 5970 | 12.0% |  | 3229 | 12.4% |  | 2178 | 11.4% |  | 563 | 12.4% |  | 2741 | 11.6% |  |
| rs3771570 | Non-case | 43753 | 88.0% |  | 28013 | 87.5% |  | 13712 | 88.7% |  | 2028 | 91.0% | <0.001 | 15740 | 89.0% | <0.001 |
|  | Case | 5957 | 12.0% |  | 4014 | 12.5% |  | 1742 | 11.3% |  | 201 | 9.0% |  | 1943 | 11.0% |  |
| rs2292884 | Non-case | 40326 | 95.9% |  | 29811 | 95.7% |  | 9708 | 96.3% |  | 807 | 95.3% | 0.034 | 10515 | 96.2% | 0.030 |
|  | Case | 1739 | 4.1% |  | 1326 | 4.3% |  | 373 | 3.7% |  | 40 | 4.7% |  | 413 | 3.8% |  |
| rs7611694 | Non-case | 38714 | 87.8% |  | 21638 | 88.2% |  | 14387 | 87.5% |  | 2689 | 86.5% | 0.006 | 17076 | 87.4% | 0.005 |
|  | Case | 5355 | 12.2% |  | 2883 | 11.8% |  | 2054 | 12.5% |  | 418 | 13.5% |  | 2472 | 12.6% |  |
| rs7758229 | Non-case | 43890 | 88.0% |  | 15025 | 87.5% |  | 20915 | 87.9% |  | 7950 | 89.3% | <0.001 | 28865 | 88.3% | 0.017 |
|  | Case | 5980 | 12.0% |  | 2141 | 12.5% |  | 2890 | 12.1% |  | 949 | 10.7% |  | 3839 | 11.7% |  |
| rs10993994 | Non-case | 43823 | 88.1% |  | 20982 | 88.7% |  | 18440 | 87.8% |  | 4401 | 86.6% | <0.001 | 22841 | 87.5% | <0.001 |
|  | Case | 5928 | 11.9% |  | 2676 | 11.3% |  | 2573 | 12.2% |  | 679 | 13.4% |  | 3252 | 12.5% |  |
| rs4430796 | Non-case | 43899 | 88.0% |  | 16275 | 89.6% |  | 20757 | 87.8% |  | 6867 | 85.3% | <0.001 | 27624 | 87.2% | <0.001 |
|  | Case | 5960 | 12.0% |  | 1897 | 10.4% |  | 2883 | 12.2% |  | 1180 | 14.7% |  | 4063 | 12.8% |  |
| rs8064454 | Non-case | 38012 | 88.0% |  | 10064 | 86.4% |  | 19146 | 88.3% |  | 8802 | 89.3% | <0.001 | 27948 | 88.6% | <0.001 |
|  | Case | 5185 | 12.0% |  | 1586 | 13.6% |  | 2540 | 11.7% |  | 1059 | 10.7% |  | 3599 | 11.4% |  |
| rs2735839 | Non-case | 43046 | 87.8% |  | 11703 | 86.3% |  | 21546 | 88.0% |  | 9797 | 89.4% | <0.001 | 31343 | 88.4% | <0.001 |
|  | Case | 5954 | 12.2% |  | 1852 | 13.7% |  | 2937 | 12.0% |  | 1165 | 10.6% |  | 4102 | 11.6% |  |

## S3 Table. Associations between selected genetic variants and prostate cancer.

| SNP | Heterozygote | |  | Rare homozygotes | |  | Per allele | |  | Heterozygote/Rare homozygotes | |
| --- | --- | --- | --- | --- | --- | --- | --- | --- | --- | --- | --- |
|  | OR(95%CI) ^*^ | P value |  | OR(95%CI) ^*^ | P value |  | OR(95%CI) ^*^ | P value |  | OR(95%CI) ^*^ | P value |
| rs11902236 | 1.05(1-1.12) | 0.070 |  | 1.09(0.99-1.2) | 0.088 |  | 1.05(1.01-1.09) | 0.028 |  | 1.06(1-1.12) | 0.034 |
| rs9306894 | 0.89(0.8-0.98) | 0.021 |  | 0.93(0.8-1.08) | 0.328 |  | 0.94(0.88-1.01) | 0.087 |  | 0.89(0.81-0.99) | 0.025 |
| rs13385191 | 0.96(0.87-1.07) | 0.488 |  | 0.99(0.81-1.2) | 0.903 |  | 0.98(0.91-1.06) | 0.605 |  | 0.97(0.88-1.07) | 0.513 |
| rs721048 | 1.1(1.04-1.18) | 0.002 |  | 1.21(1.04-1.42) | 0.016 |  | 1.1(1.05-1.16) | <0.001 |  | 1.12(1.05-1.19) | <0.001 |
| rs699664 | 0.96(0.91-1.02) | 0.210 |  | 0.98(0.89-1.07) | 0.617 |  | 0.98(0.94-1.02) | 0.340 |  | 0.97(0.92-1.02) | 0.222 |
| rs11691517 | 0.93(0.84-1.03) | 0.188 |  | 0.91(0.73-1.12) | 0.357 |  | 0.94(0.87-1.02) | 0.145 |  | 0.93(0.84-1.03) | 0.144 |
| rs6431219 | 1.04(0.98-1.11) | 0.187 |  | 0.99(0.91-1.08) | 0.762 |  | 1(0.96-1.05) | 0.893 |  | 1.03(0.97-1.09) | 0.350 |
| rs12621278 | 0.83(0.71-0.97) | 0.020 |  | 1.15(0.61-2.17) | 0.675 |  | 0.86(0.75-1) | 0.049 |  | 0.84(0.72-0.98) | 0.029 |
| rs12621900 | 1.03(0.98-1.1) | 0.242 |  | 0.99(0.88-1.11) | 0.847 |  | 1.01(0.97-1.06) | 0.531 |  | 1.03(0.97-1.09) | 0.324 |
| rs7584330 | 1.03(0.97-1.09) | 0.348 |  | 1.24(1.12-1.37) | <0.001 |  | 1.08(1.03-1.12) | 0.001 |  | 1.06(1.01-1.12) | 0.029 |
| rs77559646 | 1.1(0.87-1.39) | 0.432 |  | 1.16(0.16-8.68) | 0.882 |  | 1.1(0.87-1.38) | 0.425 |  | 1.1(0.87-1.39) | 0.426 |
| rs7618603 | 1.05(0.99-1.12) | 0.084 |  | 1.26(1.11-1.45) | 0.001 |  | 1.08(1.03-1.14) | 0.001 |  | 1.08(1.02-1.14) | 0.011 |
| rs17023900 | 1.14(1.06-1.23) | 0.001 |  | 1.45(1.1-1.92) | 0.008 |  | 1.15(1.08-1.23) | <0.001 |  | 1.15(1.07-1.24) | <0.001 |
| rs71277158 | 0.9(0.84-0.96) | 0.001 |  | 0.62(0.5-0.76) | <0.001 |  | 0.87(0.82-0.91) | <0.001 |  | 0.87(0.82-0.93) | <0.001 |
| rs78943174 | 1.09(0.92-1.3) | 0.326 |  | 2.04(0.42-9.82) | 0.374 |  | 1.1(0.93-1.31) | 0.261 |  | 1.1(0.92-1.31) | 0.291 |
| rs10009409 | 1.08(1.02-1.14) | 0.012 |  | 1.09(1-1.2) | 0.054 |  | 1.06(1.01-1.1) | 0.008 |  | 1.08(1.02-1.14) | 0.006 |
| rs1894292 | 0.96(0.9-1.02) | 0.223 |  | 0.92(0.85-1) | 0.041 |  | 0.96(0.92-1) | 0.040 |  | 0.95(0.89-1.01) | 0.088 |
| rs12500426 | 1.05(0.94-1.18) | 0.385 |  | 1.08(0.94-1.24) | 0.272 |  | 1.04(0.97-1.11) | 0.258 |  | 1.06(0.95-1.18) | 0.285 |
| rs17021918 | 0.88(0.79-0.97) | 0.013 |  | 0.75(0.63-0.89) | 0.001 |  | 0.87(0.81-0.94) | <0.001 |  | 0.85(0.77-0.94) | 0.001 |
| rs7679673 | 0.8(0.75-0.85) | <0.001 |  | 0.77(0.71-0.83) | <0.001 |  | 0.86(0.83-0.9) | <0.001 |  | 0.79(0.74-0.83) | <0.001 |
| rs6820205 | 0.95(0.87-1.03) | 0.193 |  | 0.85(0.57-1.24) | 0.394 |  | 0.94(0.87-1.02) | 0.130 |  | 0.94(0.87-1.02) | 0.152 |
| rs10069690 | 0.88(0.83-0.93) | <0.001 |  | 0.85(0.77-0.95) | 0.004 |  | 0.9(0.86-0.94) | <0.001 |  | 0.88(0.83-0.92) | <0.001 |
| rs7725218 | 0.86(0.81-0.91) | <0.001 |  | 0.73(0.67-0.8) | <0.001 |  | 0.86(0.82-0.89) | <0.001 |  | 0.83(0.79-0.88) | <0.001 |
| rs71595003 | 0.93(0.74-1.17) | 0.523 |  | 2.44(0.57-10.49) | 0.230 |  | 0.96(0.77-1.2) | 0.704 |  | 0.94(0.75-1.18) | 0.607 |
| rs4466137 | 0.92(0.83-1.02) | 0.126 |  | 1.02(0.83-1.25) | 0.874 |  | 0.96(0.89-1.04) | 0.368 |  | 0.94(0.85-1.03) | 0.185 |
| rs35148638 | 0.93(0.88-0.99) | 0.028 |  | 1.05(0.94-1.18) | 0.363 |  | 0.98(0.94-1.03) | 0.427 |  | 0.95(0.9-1.01) | 0.093 |
| rs76551843 | 0.35(0.18-0.67) | 0.002 |  | - | 0.999 |  | 0.34(0.18-0.66) | 0.001 |  | 0.34(0.18-0.66) | 0.001 |
| rs6869841 | 1.16(1.05-1.28) | 0.005 |  | 1.47(1.2-1.8) | <0.001 |  | 1.19(1.1-1.28) | <0.001 |  | 1.2(1.09-1.32) | <0.001 |
| rs4976790 | 1.02(0.96-1.09) | 0.497 |  | 1.3(1.08-1.56) | 0.006 |  | 1.06(1-1.12) | 0.051 |  | 1.04(0.98-1.11) | 0.186 |
| rs4710983 | 1(0.94-1.06) | 0.984 |  | 0.92(0.8-1.05) | 0.208 |  | 0.98(0.93-1.03) | 0.411 |  | 0.99(0.93-1.05) | 0.675 |
| rs7767188 | 1.02(0.92-1.14) | 0.668 |  | 1.03(0.81-1.32) | 0.803 |  | 1.02(0.94-1.11) | 0.645 |  | 1.02(0.93-1.13) | 0.642 |
| rs12665339 | 1.05(0.99-1.12) | 0.108 |  | 0.99(0.83-1.18) | 0.932 |  | 1.03(0.98-1.09) | 0.223 |  | 1.05(0.99-1.12) | 0.137 |
| rs6457327 | 1.08(1.02-1.15) | 0.010 |  | 1.06(0.98-1.15) | 0.162 |  | 1.04(1-1.08) | 0.043 |  | 1.08(1.02-1.14) | 0.010 |
| rs130067 | 0.99(0.93-1.06) | 0.810 |  | 1.02(0.89-1.18) | 0.762 |  | 1(0.95-1.05) | 0.994 |  | 1(0.94-1.06) | 0.893 |
| rs3096702 | 1.03(0.97-1.09) | 0.357 |  | 1.05(0.96-1.14) | 0.330 |  | 1.02(0.98-1.07) | 0.256 |  | 1.03(0.97-1.09) | 0.272 |
| rs377763 | 0.98(0.92-1.03) | 0.403 |  | 1.03(0.9-1.17) | 0.657 |  | 0.99(0.95-1.04) | 0.740 |  | 0.98(0.93-1.04) | 0.522 |
| rs9296068 | 0.98(0.93-1.04) | 0.592 |  | 0.93(0.85-1.01) | 0.094 |  | 0.97(0.93-1.01) | 0.126 |  | 0.97(0.92-1.03) | 0.301 |
| rs10498792 | 1.03(0.96-1.1) | 0.382 |  | 0.97(0.77-1.23) | 0.810 |  | 1.02(0.96-1.08) | 0.523 |  | 1.03(0.96-1.1) | 0.432 |
| rs4646284 | 1.11(1-1.22) | 0.050 |  | 1.21(1.02-1.43) | 0.029 |  | 1.1(1.02-1.18) | 0.010 |  | 1.12(1.02-1.24) | 0.017 |
| rs17621345 | 0.96(0.9-1.01) | 0.123 |  | 0.98(0.87-1.1) | 0.742 |  | 0.97(0.93-1.02) | 0.226 |  | 0.96(0.91-1.01) | 0.139 |
| rs6955627 | 0.99(0.92-1.06) | 0.740 |  | 1.06(0.86-1.29) | 0.593 |  | 1(0.94-1.06) | 0.989 |  | 0.99(0.93-1.06) | 0.866 |
| rs2572375 | 0.78(0.7-0.86) | <0.001 |  | 0.71(0.58-0.87) | 0.001 |  | 0.81(0.75-0.88) | <0.001 |  | 0.77(0.69-0.84) | <0.001 |
| rs1016343 | 1.24(1.17-1.31) | <0.001 |  | 1.58(1.4-1.79) | <0.001 |  | 1.25(1.19-1.31) | <0.001 |  | 1.28(1.21-1.35) | <0.001 |
| rs16902094 | 1.09(0.98-1.22) | 0.123 |  | 0.96(0.68-1.37) | 0.834 |  | 1.06(0.96-1.17) | 0.242 |  | 1.08(0.97-1.2) | 0.156 |
| rs445114 | 0.9(0.84-0.95) | <0.001 |  | 0.8(0.74-0.88) | <0.001 |  | 0.9(0.86-0.93) | <0.001 |  | 0.87(0.83-0.92) | <0.001 |
| rs62516032 | 0.91(0.86-0.96) | 0.001 |  | 0.83(0.75-0.91) | <0.001 |  | 0.91(0.87-0.95) | <0.001 |  | 0.89(0.84-0.94) | <0.001 |
| rs16902147 | 1.31(1.18-1.45) | <0.001 |  | 1.47(0.91-2.37) | 0.118 |  | 1.29(1.17-1.42) | <0.001 |  | 1.31(1.19-1.46) | <0.001 |
| rs10505477 | 0.78(0.74-0.84) | <0.001 |  | 0.66(0.61-0.71) | <0.001 |  | 0.81(0.78-0.84) | <0.001 |  | 0.74(0.7-0.79) | <0.001 |
| rs4242384 | 1.41(1.32-1.51) | <0.001 |  | 1.94(1.57-2.39) | <0.001 |  | 1.41(1.33-1.49) | <0.001 |  | 1.44(1.35-1.53) | <0.001 |
| rs3217992 | 0.97(0.91-1.03) | 0.285 |  | 0.94(0.86-1.03) | 0.182 |  | 0.97(0.93-1.01) | 0.143 |  | 0.96(0.91-1.02) | 0.178 |
| rs17694493 | 1.04(0.92-1.16) | 0.549 |  | 1.24(0.89-1.73) | 0.207 |  | 1.06(0.96-1.17) | 0.265 |  | 1.05(0.94-1.17) | 0.386 |
| rs817826 | 0.98(0.92-1.04) | 0.459 |  | 1.11(0.93-1.32) | 0.244 |  | 1(0.95-1.06) | 0.994 |  | 0.99(0.93-1.05) | 0.684 |
| rs4749884 | 0.92(0.87-0.98) | 0.009 |  | 0.92(0.85-1) | 0.051 |  | 0.95(0.92-0.99) | 0.015 |  | 0.92(0.87-0.98) | 0.005 |
| rs76934034 | 0.7(0.6-0.82) | <0.001 |  | 1.17(0.68-2) | 0.576 |  | 0.76(0.66-0.88) | <0.001 |  | 0.72(0.62-0.84) | <0.001 |
| rs3123078 | 1.14(1.07-1.21) | <0.001 |  | 1.4(1.29-1.51) | <0.001 |  | 1.18(1.13-1.22) | <0.001 |  | 1.21(1.14-1.28) | <0.001 |
| rs4554825 | 0.97(0.89-1.05) | 0.416 |  | 0.83(0.57-1.21) | 0.331 |  | 0.96(0.89-1.03) | 0.258 |  | 0.96(0.88-1.04) | 0.322 |
| rs1935581 | 0.95(0.89-1.01) | 0.120 |  | 0.95(0.87-1.04) | 0.291 |  | 0.97(0.93-1.01) | 0.171 |  | 0.95(0.9-1.01) | 0.105 |
| rs3850699 | 0.96(0.9-1.02) | 0.153 |  | 0.82(0.74-0.92) | 0.001 |  | 0.93(0.89-0.97) | 0.001 |  | 0.93(0.88-0.99) | 0.019 |
| rs2252004 | 1.3(1.16-1.46) | <0.001 |  | 3.17(2.58-3.9) | <0.001 |  | 1.53(1.4-1.66) | <0.001 |  | 1.49(1.34-1.65) | <0.001 |
| rs61890184 | 1.1(1.03-1.17) | 0.006 |  | 1.07(0.86-1.32) | 0.538 |  | 1.08(1.02-1.14) | 0.009 |  | 1.09(1.03-1.16) | 0.005 |
| rs11214775 | 0.94(0.89-1) | 0.042 |  | 0.85(0.77-0.95) | 0.003 |  | 0.93(0.89-0.97) | 0.001 |  | 0.93(0.88-0.98) | 0.006 |
| rs2066827 | 1.02(0.96-1.09) | 0.509 |  | 1.01(0.9-1.13) | 0.919 |  | 1.01(0.97-1.06) | 0.636 |  | 1.02(0.96-1.08) | 0.538 |
| rs1635553 | 0.98(0.92-1.05) | 0.604 |  | 0.99(0.91-1.07) | 0.774 |  | 0.99(0.95-1.03) | 0.741 |  | 0.98(0.92-1.05) | 0.620 |
| rs10875943 | 1.19(1.07-1.32) | 0.001 |  | 1.85(1.62-2.12) | <0.001 |  | 1.33(1.24-1.42) | <0.001 |  | 1.34(1.21-1.47) | <0.001 |
| rs12322335 | 1.08(1.02-1.15) | 0.008 |  | 1.17(1.02-1.34) | 0.024 |  | 1.08(1.03-1.14) | 0.001 |  | 1.09(1.03-1.16) | 0.002 |
| rs902774 | 1.19(1.12-1.27) | <0.001 |  | 1.24(1.03-1.48) | 0.021 |  | 1.17(1.11-1.23) | <0.001 |  | 1.2(1.13-1.27) | <0.001 |
| rs10774740 | 0.89(0.84-0.95) | <0.001 |  | 0.85(0.78-0.92) | <0.001 |  | 0.92(0.88-0.95) | <0.001 |  | 0.88(0.83-0.93) | <0.001 |
| rs1270884 | 0.76(0.68-0.85) | <0.001 |  | 0.78(0.68-0.89) | <0.001 |  | 0.87(0.81-0.93) | <0.001 |  | 0.77(0.69-0.85) | <0.001 |
| rs9600079 | 0.99(0.93-1.06) | 0.874 |  | 0.98(0.9-1.06) | 0.571 |  | 0.99(0.95-1.03) | 0.589 |  | 0.99(0.93-1.05) | 0.737 |
| rs17522122 | 0.85(0.77-0.95) | 0.005 |  | 0.7(0.6-0.81) | <0.001 |  | 0.84(0.78-0.9) | <0.001 |  | 0.81(0.73-0.9) | <0.001 |
| rs8008270 | 0.97(0.91-1.03) | 0.285 |  | 0.95(0.82-1.09) | 0.455 |  | 0.97(0.92-1.02) | 0.218 |  | 0.97(0.91-1.02) | 0.230 |
| rs7153648 | 1.48(1.32-1.66) | <0.001 |  | 3.04(2.3-4.02) | <0.001 |  | 1.57(1.43-1.73) | <0.001 |  | 1.58(1.42-1.77) | <0.001 |
| rs7141529 | 0.96(0.9-1.03) | 0.254 |  | 0.98(0.91-1.05) | 0.564 |  | 0.99(0.95-1.03) | 0.536 |  | 0.97(0.91-1.03) | 0.293 |
| rs8014671 | 0.96(0.9-1.02) | 0.162 |  | 0.92(0.85-1) | 0.045 |  | 0.96(0.92-1) | 0.038 |  | 0.95(0.9-1) | 0.066 |
| rs4775302 | 1.07(0.95-1.2) | 0.264 |  | 1.14(0.99-1.3) | 0.061 |  | 1.07(1-1.14) | 0.061 |  | 1.09(0.98-1.22) | 0.119 |
| rs33984059 | 0.71(0.54-0.93) | 0.015 |  | - | 0.998 |  | 0.69(0.53-0.91) | 0.009 |  | 0.7(0.53-0.92) | 0.011 |
| rs13380763 | 0.97(0.91-1.03) | 0.260 |  | 0.92(0.79-1.07) | 0.285 |  | 0.96(0.92-1.01) | 0.143 |  | 0.96(0.91-1.02) | 0.180 |
| rs684232 | 1.04(0.98-1.1) | 0.227 |  | 1.23(1.14-1.34) | <0.001 |  | 1.1(1.05-1.14) | <0.001 |  | 1.08(1.02-1.15) | 0.005 |
| rs78378222 | 1.57(1.2-2.07) | 0.001 |  | 0(0-0) | 0.999 |  | 1.54(1.17-2.01) | 0.002 |  | 1.56(1.19-2.05) | 0.001 |
| rs7501939 | 0.88(0.83-0.93) | <0.001 |  | 0.81(0.74-0.88) | <0.001 |  | 0.9(0.86-0.93) | <0.001 |  | 0.86(0.82-0.91) | <0.001 |
| rs138213197 | 4.31(2.7-6.88) | <0.001 |  | - |  |  | 4.31(2.7-6.88) | <0.001 |  | 4.31(2.7-6.88) | <0.001 |
| rs11650494 | 1.17(1.09-1.26) | <0.001 |  | 0.97(0.7-1.33) | 0.850 |  | 1.14(1.06-1.22) | <0.001 |  | 1.16(1.08-1.25) | <0.001 |
| rs7210100 | 1.7(1.19-2.44) | 0.004 |  | 7.37(1.04-52.3) | 0.046 |  | 1.79(1.28-2.51) | 0.001 |  | 1.77(1.25-2.52) | 0.001 |
| rs2680708 | 0.98(0.92-1.04) | 0.517 |  | 1.01(0.93-1.09) | 0.848 |  | 1(0.96-1.04) | 0.979 |  | 0.99(0.93-1.04) | 0.661 |
| rs17765344 | 1.04(0.97-1.11) | 0.297 |  | 1.27(1.18-1.37) | <0.001 |  | 1.13(1.08-1.17) | <0.001 |  | 1.11(1.04-1.18) | 0.001 |
| rs7236466 | 1.02(0.96-1.08) | 0.608 |  | 1.04(0.96-1.12) | 0.376 |  | 1.02(0.98-1.06) | 0.372 |  | 1.02(0.97-1.08) | 0.467 |
| rs7241993 | 0.97(0.92-1.03) | 0.334 |  | 0.98(0.89-1.08) | 0.675 |  | 0.98(0.94-1.02) | 0.426 |  | 0.97(0.92-1.03) | 0.335 |
| rs12610088 | 0.97(0.91-1.03) | 0.251 |  | 1.02(0.94-1.1) | 0.710 |  | 1(0.96-1.04) | 0.950 |  | 0.98(0.92-1.03) | 0.439 |
| rs61088131 | 1.24(1.11-1.38) | <0.001 |  | 2.4(2.03-2.83) | <0.001 |  | 1.43(1.32-1.54) | <0.001 |  | 1.41(1.28-1.55) | <0.001 |
| rs2659051 | 0.93(0.84-1.03) | 0.148 |  | 0.71(0.55-0.92) | 0.009 |  | 0.89(0.82-0.97) | 0.008 |  | 0.9(0.81-0.99) | 0.036 |
| rs2659124 | 1.01(0.91-1.13) | 0.823 |  | 0.68(0.48-0.96) | 0.027 |  | 0.95(0.87-1.04) | 0.290 |  | 0.98(0.88-1.09) | 0.694 |
| rs62113212 | 0.68(0.58-0.8) | <0.001 |  | 0.25(0.08-0.77) | 0.016 |  | 0.66(0.56-0.77) | <0.001 |  | 0.66(0.56-0.77) | <0.001 |
| rs61752561 | 0.99(0.89-1.1) | 0.807 |  | 0.67(0.31-1.47) | 0.320 |  | 0.97(0.88-1.08) | 0.601 |  | 0.98(0.88-1.09) | 0.700 |
| rs17632542 | 0.69(0.59-0.81) | <0.001 |  | 0.25(0.08-0.78) | 0.017 |  | 0.67(0.57-0.78) | <0.001 |  | 0.67(0.57-0.78) | <0.001 |
| rs103294 | 0.97(0.91-1.03) | 0.293 |  | 0.92(0.81-1.04) | 0.191 |  | 0.96(0.92-1.01) | 0.123 |  | 0.96(0.9-1.02) | 0.174 |
| rs7258285 | 0.99(0.93-1.05) | 0.709 |  | 0.99(0.91-1.08) | 0.880 |  | 0.99(0.96-1.03) | 0.795 |  | 0.99(0.94-1.05) | 0.722 |
| rs73140002 | 1.07(0.96-1.2) | 0.211 |  | 1.06(0.58-1.95) | 0.849 |  | 1.07(0.96-1.18) | 0.215 |  | 1.07(0.96-1.19) | 0.206 |
| rs2427345 | 1.01(0.95-1.07) | 0.808 |  | 1.05(0.97-1.14) | 0.247 |  | 1.02(0.98-1.06) | 0.308 |  | 1.02(0.96-1.08) | 0.541 |
| rs6062509 | 0.81(0.73-0.89) | <0.001 |  | 0.72(0.6-0.86) | <0.001 |  | 0.83(0.77-0.89) | <0.001 |  | 0.79(0.72-0.87) | <0.001 |
| rs2823739 | 1(0.92-1.08) | 0.991 |  | 0.93(0.65-1.33) | 0.697 |  | 0.99(0.92-1.07) | 0.887 |  | 1(0.92-1.08) | 0.949 |
| rs1041449 | 1.09(1.02-1.16) | 0.013 |  | 1.17(1.08-1.27) | <0.001 |  | 1.08(1.04-1.13) | <0.001 |  | 1.11(1.04-1.18) | 0.001 |
| rs138708 | 0.85(0.74-0.98) | 0.022 |  | 1.16(0.52-2.59) | 0.712 |  | 0.87(0.76-0.99) | 0.038 |  | 0.86(0.75-0.98) | 0.027 |
| rs9623117 | 1.01(0.91-1.13) | 0.813 |  | 2.23(1.93-2.59) | <0.001 |  | 1.33(1.23-1.43) | <0.001 |  | 1.21(1.1-1.34) | <0.001 |
| rs5759167 | 0.87(0.82-0.92) | <0.001 |  | 0.76(0.7-0.82) | <0.001 |  | 0.87(0.84-0.9) | <0.001 |  | 0.83(0.78-0.88) | <0.001 |
| rs2405942 | - | 0.999 |  | 1(0.94-1.07) | 0.995 |  | 1(0.97-1.03) | 0.990 |  | 1(0.93-1.07) | 0.980 |
| rs2788524 | 1.78(0.23-13.64) | 0.577 |  | 0.83(0.71-0.96) | 0.010 |  | 0.91(0.85-0.98) | 0.011 |  | 0.83(0.72-0.96) | 0.011 |
| rs5945572 | - | 0.999 |  | 1.19(1.12-1.26) | <0.001 |  | 1.09(1.06-1.12) | <0.001 |  | 1.19(1.12-1.26) | <0.001 |
| rs2807031 | - | 0.999 |  | 1.05(0.93-1.19) | 0.408 |  | 1.03(0.96-1.09) | 0.410 |  | 1.05(0.93-1.19) | 0.413 |
| rs5919432 | - | 0.999 |  | 0.97(0.9-1.03) | 0.302 |  | 0.98(0.95-1.02) | 0.300 |  | 0.96(0.9-1.03) | 0.295 |
| rs6625711 | - | - |  | - | - |  | - | - |  | - | - |
| rs10503733 | 1.07(1.01-1.14) | 0.023 |  | 1.12(1-1.26) | 0.058 |  | 1.07(1.02-1.12) | 0.007 |  | 1.08(1.02-1.14) | 0.009 |
| rs636291 | 1.12(1.01-1.24) | 0.034 |  | 1.63(1.42-1.88) | <0.001 |  | 1.24(1.16-1.33) | <0.001 |  | 1.23(1.11-1.35) | <0.001 |
| rs17599629 | 1.11(1.05-1.17) | <0.001 |  | 1.2(1.06-1.36) | 0.005 |  | 1.1(1.05-1.15) | <0.001 |  | 1.12(1.06-1.18) | <0.001 |
| rs4245739 | 0.91(0.86-0.97) | 0.002 |  | 0.95(0.85-1.06) | 0.339 |  | 0.94(0.9-0.99) | 0.010 |  | 0.92(0.87-0.97) | 0.002 |
| rs9287719 | 1.01(0.9-1.13) | 0.863 |  | 0.98(0.85-1.12) | 0.728 |  | 0.99(0.92-1.06) | 0.763 |  | 1(0.9-1.11) | 0.994 |
| rs77482050 | 0.89(0.7-1.12) | 0.322 |  | - | 0.999 |  | 0.88(0.7-1.11) | 0.287 |  | 0.88(0.7-1.12) | 0.303 |
| rs9284813 | 1.07(1-1.13) | 0.047 |  | 1.33(1.14-1.55) | <0.001 |  | 1.1(1.04-1.16) | <0.001 |  | 1.09(1.03-1.16) | 0.005 |
| rs17181170 | 0.93(0.88-0.99) | 0.033 |  | 0.83(0.77-0.9) | <0.001 |  | 0.91(0.88-0.95) | <0.001 |  | 0.9(0.85-0.96) | <0.001 |
| rs6808932 | 0.94(0.89-1) | 0.062 |  | 0.88(0.81-0.96) | 0.002 |  | 0.94(0.9-0.98) | 0.002 |  | 0.93(0.88-0.98) | 0.009 |
| rs10934853 | 1.1(1.04-1.16) | 0.001 |  | 1.15(1.05-1.27) | 0.002 |  | 1.08(1.04-1.13) | <0.001 |  | 1.11(1.05-1.17) | <0.001 |
| rs6763931 | 1.05(0.99-1.12) | 0.128 |  | 1.14(1.05-1.23) | 0.001 |  | 1.07(1.03-1.11) | 0.001 |  | 1.08(1.01-1.14) | 0.015 |
| rs12653946 | 1.14(1.08-1.22) | <0.001 |  | 1.22(1.13-1.32) | <0.001 |  | 1.11(1.07-1.15) | <0.001 |  | 1.16(1.1-1.23) | <0.001 |
| rs2121875 | 1.04(0.99-1.11) | 0.141 |  | 1.19(1.09-1.29) | <0.001 |  | 1.08(1.04-1.12) | <0.001 |  | 1.08(1.02-1.14) | 0.008 |
| rs1983891 | 1.25(1.13-1.38) | <0.001 |  | 1.72(1.47-2.01) | <0.001 |  | 1.29(1.2-1.39) | <0.001 |  | 1.33(1.2-1.46) | <0.001 |
| rs2273669 | 1.05(0.99-1.12) | 0.123 |  | 1.22(1.03-1.45) | 0.021 |  | 1.07(1.01-1.13) | 0.014 |  | 1.07(1-1.13) | 0.041 |
| rs339331 | 0.92(0.86-0.97) | 0.002 |  | 0.82(0.74-0.9) | <0.001 |  | 0.91(0.87-0.95) | <0.001 |  | 0.9(0.85-0.95) | <0.001 |
| rs1933488 | 0.95(0.89-1.01) | 0.083 |  | 0.86(0.8-0.93) | <0.001 |  | 0.93(0.9-0.97) | <0.001 |  | 0.92(0.87-0.98) | 0.006 |
| rs9364554 | 1.12(1.05-1.18) | <0.001 |  | 1.26(1.14-1.39) | <0.001 |  | 1.12(1.07-1.17) | <0.001 |  | 1.14(1.08-1.2) | <0.001 |
| rs12155172 | 1.08(1.02-1.14) | 0.013 |  | 1.17(1.04-1.32) | 0.011 |  | 1.08(1.03-1.13) | 0.001 |  | 1.09(1.03-1.15) | 0.003 |
| rs6465657 | 1.08(1.01-1.16) | 0.017 |  | 1.14(1.06-1.23) | 0.001 |  | 1.07(1.03-1.11) | 0.001 |  | 1.1(1.04-1.17) | 0.002 |
| rs1512268 | 1.17(1.09-1.25) | <0.001 |  | 1.3(1.19-1.41) | <0.001 |  | 1.14(1.1-1.19) | <0.001 |  | 1.2(1.13-1.28) | <0.001 |
| rs11135910 | 1.12(1.05-1.19) | <0.001 |  | 1.07(0.9-1.28) | 0.427 |  | 1.09(1.04-1.15) | 0.001 |  | 1.11(1.05-1.18) | <0.001 |
| rs1456315 | 1.08(1.02-1.14) | 0.013 |  | 1.24(1.14-1.35) | <0.001 |  | 1.1(1.06-1.15) | <0.001 |  | 1.11(1.05-1.18) | <0.001 |
| rs10505483 | 1.41(1.29-1.54) | <0.001 |  | 1.95(1.52-2.49) | <0.001 |  | 1.4(1.31-1.51) | <0.001 |  | 1.45(1.34-1.58) | <0.001 |
| rs188140481 | 1.87(1.22-2.86) | 0.004 |  | - | 0.999 |  | 2.16(1.46-3.21) | <0.001 |  | 2.03(1.34-3.06) | 0.001 |
| rs11199874 | 0.89(0.8-0.99) | 0.027 |  | 0.83(0.67-1.03) | 0.085 |  | 0.9(0.83-0.97) | 0.010 |  | 0.88(0.8-0.97) | 0.012 |
| rs4962416 | 1.11(1.05-1.17) | <0.001 |  | 1.13(1.02-1.25) | 0.022 |  | 1.08(1.04-1.13) | <0.001 |  | 1.11(1.05-1.17) | <0.001 |
| rs7126629 | 1.28(1.16-1.42) | <0.001 |  | 1.58(1.28-1.96) | <0.001 |  | 1.27(1.17-1.38) | <0.001 |  | 1.32(1.19-1.45) | <0.001 |
| rs11228565 | 1.15(1.08-1.22) | <0.001 |  | 1.33(1.17-1.51) | <0.001 |  | 1.15(1.1-1.2) | <0.001 |  | 1.17(1.1-1.23) | <0.001 |
| rs7929962 | 1.21(1.13-1.29) | <0.001 |  | 1.43(1.32-1.54) | <0.001 |  | 1.19(1.15-1.24) | <0.001 |  | 1.28(1.2-1.37) | <0.001 |
| rs7130881 | 1.26(1.19-1.34) | <0.001 |  | 1.48(1.27-1.72) | <0.001 |  | 1.24(1.18-1.31) | <0.001 |  | 1.28(1.21-1.36) | <0.001 |
| rs11568818 | 1.03(0.97-1.1) | 0.301 |  | 0.94(0.86-1.02) | 0.118 |  | 0.98(0.94-1.02) | 0.247 |  | 1.01(0.95-1.07) | 0.853 |
| rs8102476 | 0.91(0.85-0.97) | 0.005 |  | 0.84(0.77-0.91) | <0.001 |  | 0.92(0.88-0.95) | <0.001 |  | 0.89(0.84-0.95) | <0.001 |
| rs11672691 | 0.91(0.86-0.97) | 0.002 |  | 1(0.91-1.1) | 0.966 |  | 0.97(0.93-1.01) | 0.097 |  | 0.93(0.88-0.98) | 0.008 |
| rs1058205 | 0.89(0.84-0.94) | <0.001 |  | 0.69(0.6-0.8) | <0.001 |  | 0.86(0.82-0.91) | <0.001 |  | 0.86(0.81-0.91) | <0.001 |
| rs3771570 | 0.86(0.77-0.97) | 0.014 |  | 1.11(0.81-1.54) | 0.510 |  | 0.92(0.83-1.01) | 0.093 |  | 0.88(0.79-0.99) | 0.030 |
| rs2292884 | 1.07(1.01-1.14) | 0.025 |  | 1.17(1.05-1.3) | 0.006 |  | 1.08(1.03-1.13) | 0.001 |  | 1.09(1.03-1.15) | 0.005 |
| rs7611694 | 0.97(0.91-1.03) | 0.312 |  | 0.84(0.77-0.91) | <0.001 |  | 0.93(0.89-0.96) | <0.001 |  | 0.93(0.88-0.99) | 0.017 |
| rs7758229 | 1.09(1.03-1.16) | 0.002 |  | 1.21(1.11-1.32) | <0.001 |  | 1.1(1.05-1.14) | <0.001 |  | 1.12(1.06-1.18) | <0.001 |
| rs10993994 | 1.19(1.12-1.27) | <0.001 |  | 1.47(1.36-1.59) | <0.001 |  | 1.21(1.17-1.26) | <0.001 |  | 1.26(1.19-1.34) | <0.001 |
| rs4430796 | 0.84(0.79-0.9) | <0.001 |  | 0.76(0.7-0.83) | <0.001 |  | 0.87(0.84-0.91) | <0.001 |  | 0.82(0.77-0.87) | <0.001 |
| rs8064454 | 0.86(0.81-0.92) | <0.001 |  | 0.75(0.7-0.81) | <0.001 |  | 0.87(0.83-0.9) | <0.001 |  | 0.83(0.78-0.88) | <0.001 |
| rs2735839 | 1.13(1.01-1.25) | 0.027 |  | 1.16(0.9-1.5) | 0.245 |  | 1.11(1.02-1.21) | 0.020 |  | 1.13(1.02-1.25) | 0.018 |

*, ORs were calculated with univariate logistics regression.

## S4 Table. Associations between per allele of genetic variants and prostate cancer after harmonizing the direction of association and imputing missing data of index SNP with common homozygotes.

| SNP | Risk alleles | Non-risk alleles | Before imputation | | |  | After imputation | | |
| --- | --- | --- | --- | --- | --- | --- | --- | --- | --- |
|  |  |  | beta^*^ | OR(95%CI) | P value |  | beta^*^ | OR(95%CI) | P value |
| rs11902236 | T | C | 0.047 | 1.05(1.01-1.09) | 0.028 |  | 0.048 | 1.05(1.01-1.09) | 0.024 |
| rs721048 | A | G | 0.099 | 1.10(1.05-1.16) | <0.001 |  | 0.117 | 1.12(1.07-1.18) | <0.001 |
| rs699664 | C | T | 0.020 | 1.02(0.98-1.06) | 0.340 |  | 0.020 | 1.02(0.98-1.06) | 0.338 |
| rs6431219 | T | C | 0.003 | 1.00(0.96-1.05) | 0.893 |  | 0.045 | 1.05(1.01-1.09) | 0.020 |
| rs12621900 | T | C | 0.014 | 1.01(0.97-1.06) | 0.531 |  | 0.010 | 1.01(0.97-1.06) | 0.659 |
| rs7584330 | G | A | 0.074 | 1.08(1.03-1.12) | 0.001 |  | 0.072 | 1.07(1.03-1.12) | 0.001 |
| rs7618603 | A | C | 0.080 | 1.08(1.03-1.14) | 0.001 |  | 0.079 | 1.08(1.03-1.13) | 0.001 |
| rs17023900 | G | A | 0.141 | 1.15(1.08-1.23) | <0.001 |  | 0.123 | 1.13(1.06-1.21) | <0.001 |
| rs71277158 | T | G | 0.145 | 1.16(1.09-1.22) | <0.001 |  | 0.211 | 1.23(1.17-1.3) | <0.001 |
| rs78943174 | T | C | 0.098 | 1.10(0.93-1.31) | 0.261 |  | 0.128 | 1.14(0.96-1.35) | 0.140 |
| rs10009409 | T | C | 0.054 | 1.06(1.01-1.10) | 0.008 |  | 0.053 | 1.05(1.01-1.10) | 0.010 |
| rs1894292 | G | A | 0.040 | 1.04(1.00-1.08) | 0.040 |  | 0.067 | 1.07(1.03-1.11) | <0.001 |
| rs7679673 | C | A | 0.150 | 1.16(1.12-1.21) | <0.001 |  | 0.088 | 1.09(1.05-1.13) | <0.001 |
| rs6820205 | C | T | 0.059 | 1.06(0.98-1.14) | 0.130 |  | 0.213 | 1.24(1.16-1.32) | <0.001 |
| rs10069690 | C | T | 0.102 | 1.11(1.06-1.16) | <0.001 |  | 0.096 | 1.10(1.05-1.15) | <0.001 |
| rs7725218 | G | A | 0.152 | 1.16(1.12-1.21) | <0.001 |  | 0.187 | 1.21(1.16-1.25) | <0.001 |
| rs35148638 | A | C | 0.019 | 1.02(0.97-1.07) | 0.427 |  | 0.042 | 1.04(1.01-1.08) | 0.022 |
| rs4976790 | T | G | 0.055 | 1.06(1.00-1.12) | 0.051 |  | 0.055 | 1.06(1.00-1.12) | 0.051 |
| rs4710983 | T | C | 0.021 | 1.02(0.97-1.07) | 0.411 |  | 0.092 | 1.10(1.06-1.14) | <0.001 |
| rs12665339 | G | A | 0.034 | 1.03(0.98-1.09) | 0.223 |  | 0.050 | 1.05(1.00-1.11) | 0.065 |
| rs6457327 | A | C | 0.040 | 1.04(1.00-1.08) | 0.043 |  | 0.040 | 1.04(1.00-1.08) | 0.044 |
| rs130067 | T | G | <0.001 | 1.00 (0.95-1.05) | 0.994 |  | 0.033 | 1.03(1.00-1.07) | 0.080 |
| rs3096702 | A | G | 0.024 | 1.02(0.98-1.07) | 0.256 |  | 0.063 | 1.07(1.02-1.11) | 0.002 |
| rs377763 | C | A | 0.008 | 1.01(0.96-1.06) | 0.740 |  | 0.006 | 1.01(0.96-1.05) | 0.807 |
| rs9296068 | T | G | 0.031 | 1.03(0.99-1.07) | 0.126 |  | 0.029 | 1.03(0.99-1.07) | 0.146 |
| rs10498792 | C | T | 0.019 | 1.02(0.96-1.08) | 0.523 |  | 0.014 | 1.01(0.96-1.08) | 0.641 |
| rs17621345 | A | C | 0.027 | 1.03(0.98-1.07) | 0.226 |  | 0.018 | 1.02(0.97-1.06) | 0.421 |
| rs1016343 | T | C | 0.222 | 1.25(1.19-1.31) | <0.001 |  | 0.221 | 1.25(1.19-1.31) | <0.001 |
| rs445114 | T | C | 0.109 | 1.12(1.07-1.16) | <0.001 |  | 0.103 | 1.11(1.07-1.15) | <0.001 |
| rs62516032 | T | C | 0.097 | 1.10(1.06-1.15) | <0.001 |  | 0.134 | 1.14(1.10-1.19) | <0.001 |
| rs16902147 | C | T | 0.257 | 1.29(1.17-1.42) | <0.001 |  | 0.266 | 1.31(1.19-1.44) | <0.001 |
| rs10505477 | A | G | 0.211 | 1.24(1.19-1.28) | <0.001 |  | 0.211 | 1.23(1.19-1.28) | <0.001 |
| rs4242384 | C | A | 0.341 | 1.41(1.33-1.49) | <0.001 |  | 0.350 | 1.42(1.34-1.50) | <0.001 |
| rs3217992 | C | T | 0.031 | 1.03(0.99-1.08) | 0.143 |  | 0.054 | 1.06(1.02-1.09) | 0.003 |
| rs4749884 | C | A | 0.048 | 1.05(1.01-1.09) | 0.015 |  | 0.048 | 1.05(1.01-1.09) | 0.015 |
| rs3123078 | C | T | 0.164 | 1.18(1.13-1.22) | <0.001 |  | 0.162 | 1.18(1.13-1.22) | <0.001 |
| rs1935581 | C | T | 0.029 | 1.03(0.99-1.07) | 0.171 |  | 0.022 | 1.02(0.99-1.06) | 0.211 |
| rs3850699 | A | G | 0.074 | 1.08(1.03-1.13) | 0.001 |  | 0.076 | 1.08(1.04-1.12) | <0.001 |
| rs61890184 | A | G | 0.076 | 1.08(1.02-1.14) | 0.009 |  | 0.091 | 1.09(1.03-1.16) | 0.002 |
| rs11214775 | G | A | 0.070 | 1.07(1.03-1.12) | 0.001 |  | 0.064 | 1.07(1.02-1.11) | 0.003 |
| rs2066827 | G | T | 0.011 | 1.01(0.97-1.06) | 0.636 |  | 0.047 | 1.05(1.00-1.10) | 0.039 |
| rs1635553 | A | G | 0.007 | 1.01(0.97-1.05) | 0.741 |  | 0.031 | 1.03(0.99-1.07) | 0.094 |
| rs12322335 | C | T | 0.080 | 1.08(1.03-1.14) | 0.001 |  | 0.078 | 1.08(1.03-1.13) | 0.001 |
| rs902774 | A | G | 0.154 | 1.17(1.11-1.23) | <0.001 |  | 0.153 | 1.17(1.11-1.23) | <0.001 |
| rs10774740 | G | T | 0.088 | 1.09(1.05-1.14) | <0.001 |  | 0.088 | 1.09(1.05-1.14) | <0.001 |
| rs8008270 | C | T | 0.031 | 1.03(0.98-1.08) | 0.218 |  | 0.031 | 1.03(0.98-1.08) | 0.220 |
| rs7141529 | C | T | 0.012 | 1.01(0.97-1.05) | 0.536 |  | 0.009 | 1.01(0.97-1.05) | 0.633 |
| rs8014671 | G | A | 0.041 | 1.04(1.00-1.08) | 0.038 |  | 0.032 | 1.03(0.99-1.07) | 0.099 |
| rs13380763 | C | T | 0.037 | 1.04(0.99-1.09) | 0.143 |  | 0.096 | 1.10(1.05-1.15) | <0.001 |
| rs684232 | C | T | 0.091 | 1.10(1.05-1.14) | <0.001 |  | 0.084 | 1.09(1.05-1.13) | <0.001 |
| rs7501939 | C | T | 0.111 | 1.12(1.07-1.16) | <0.001 |  | 0.072 | 1.07(1.03-1.12) | <0.001 |
| rs138213197 | T | C | 1.460 | 4.31(2.70-6.88) | <0.001 |  | 0.302 | 1.35(0.85-2.16) | 0.204 |
| rs11650494 | A | G | 0.129 | 1.14(1.06-1.22) | <0.001 |  | 0.143 | 1.15(1.08-1.23) | <0.001 |
| rs7210100 | A | G | 0.584 | 1.79(1.28-2.51) | 0.001 |  | 0.583 | 1.79(1.28-2.50) | 0.001 |
| rs2680708 | A | G | 0.001 | 1.00 (0.96-1.04) | 0.979 |  | 0.033 | 1.03(0.99-1.07) | 0.090 |
| rs17765344 | A | G | 0.119 | 1.13(1.08-1.17) | <0.001 |  | 0.097 | 1.10(1.06-1.14) | <0.001 |
| rs7236466 | G | T | 0.018 | 1.02(0.98-1.06) | 0.372 |  | 0.017 | 1.02(0.98-1.06) | 0.382 |
| rs61752561 | G | A | 0.027 | 1.03(0.93-1.14) | 0.601 |  | 0.233 | 1.26(1.16-1.37) | <0.001 |
| rs103294 | T | C | 0.038 | 1.04(0.99-1.09) | 0.123 |  | 0.058 | 1.06(1.02-1.10) | 0.002 |
| rs7258285 | G | A | 0.005 | 1.01(0.97-1.05) | 0.795 |  | 0.003 | 1.00 (0.96-1.04) | 0.868 |
| rs73140002 | C | T | 0.065 | 1.07(0.96-1.18) | 0.215 |  | 0.095 | 1.10(0.99-1.22) | 0.071 |
| rs2427345 | T | C | 0.020 | 1.02(0.98-1.06) | 0.308 |  | 0.017 | 1.02(0.98-1.06) | 0.406 |
| rs1041449 | G | A | 0.079 | 1.08(1.04-1.13) | <0.001 |  | 0.114 | 1.12(1.08-1.16) | <0.001 |
| rs138708 | G | A | 0.142 | 1.15(1.01-1.32) | 0.038 |  | 0.415 | 1.51(1.38-1.66) | <0.001 |
| rs5759167 | G | T | 0.140 | 1.15(1.11-1.20) | <0.001 |  | 0.140 | 1.15(1.11-1.19) | <0.001 |
| rs5945572 | A | G | 0.086 | 1.09(1.06-1.12) | <0.001 |  | 0.092 | 1.10(1.07-1.13) | <0.001 |
| rs5919432 | T | C | 0.018 | 1.02(0.98-1.05) | 0.300 |  | 0.056 | 1.06(1.02-1.09) | 0.001 |
| rs10503733 | T | G | 0.063 | 1.07(1.02-1.12) | 0.007 |  | 0.077 | 1.08(1.03-1.13) | 0.001 |
| rs17599629 | G | A | 0.097 | 1.10(1.05-1.15) | <0.001 |  | 0.099 | 1.10(1.05-1.16) | <0.001 |
| rs4245739 | A | C | 0.057 | 1.06(1.01-1.11) | 0.010 |  | 0.053 | 1.05(1.01-1.10) | 0.017 |
| rs77482050 | G | A | 0.126 | 1.13(0.90-1.43) | 0.287 |  | 0.515 | 1.67(1.45-1.93) | <0.001 |
| rs9284813 | G | A | 0.092 | 1.10(1.04-1.16) | <0.001 |  | 0.106 | 1.11(1.06-1.17) | <0.001 |
| rs17181170 | G | A | 0.092 | 1.10(1.06-1.14) | <0.001 |  | 0.091 | 1.10(1.05-1.14) | <0.001 |
| rs6808932 | A | C | 0.062 | 1.06(1.02-1.11) | 0.002 |  | 0.058 | 1.06(1.02-1.10) | 0.003 |
| rs10934853 | A | C | 0.080 | 1.08(1.04-1.13) | <0.001 |  | 0.080 | 1.08(1.04-1.13) | <0.001 |
| rs6763931 | A | G | 0.063 | 1.07(1.03-1.11) | 0.001 |  | 0.062 | 1.06(1.02-1.10) | 0.001 |
| rs12653946 | T | C | 0.102 | 1.11(1.07-1.15) | <0.001 |  | 0.100 | 1.11(1.06-1.15) | <0.001 |
| rs2121875 | C | A | 0.076 | 1.08(1.04-1.12) | <0.001 |  | 0.069 | 1.07(1.03-1.11) | 0.001 |
| rs2273669 | G | A | 0.066 | 1.07(1.01-1.13) | 0.014 |  | 0.057 | 1.06(1.00-1.12) | 0.035 |
| rs339331 | T | C | 0.096 | 1.10(1.06-1.15) | <0.001 |  | 0.051 | 1.05(1.01-1.10) | 0.016 |
| rs1933488 | A | G | 0.070 | 1.07(1.03-1.11) | <0.001 |  | 0.068 | 1.07(1.03-1.11) | 0.001 |
| rs9364554 | T | C | 0.113 | 1.12(1.07-1.17) | <0.001 |  | 0.103 | 1.11(1.06-1.16) | <0.001 |
| rs12155172 | A | G | 0.076 | 1.08(1.03-1.13) | 0.001 |  | 0.069 | 1.07(1.02-1.12) | 0.003 |
| rs6465657 | C | T | 0.066 | 1.07(1.03-1.11) | 0.001 |  | 0.063 | 1.07(1.03-1.11) | 0.001 |
| rs1512268 | T | C | 0.132 | 1.14(1.10-1.19) | <0.001 |  | 0.126 | 1.13(1.09-1.18) | <0.001 |
| rs11135910 | T | C | 0.087 | 1.09(1.04-1.15) | 0.001 |  | 0.086 | 1.09(1.04-1.15) | 0.001 |
| rs1456315 | T | C | 0.100 | 1.10(1.06-1.15) | <0.001 |  | 0.097 | 1.10(1.06-1.15) | <0.001 |
| rs10505483 | T | C | 0.339 | 1.40(1.31-1.51) | <0.001 |  | 0.338 | 1.40(1.31-1.51) | <0.001 |
| rs4962416 | C | T | 0.078 | 1.08(1.04-1.13) | <0.001 |  | 0.074 | 1.08(1.03-1.12) | 0.001 |
| rs11228565 | A | G | 0.139 | 1.15(1.10-1.20) | <0.001 |  | 0.139 | 1.15(1.10-1.20) | <0.001 |
| rs7929962 | T | C | 0.177 | 1.19(1.15-1.24) | <0.001 |  | 0.176 | 1.19(1.15-1.24) | <0.001 |
| rs7130881 | G | A | 0.219 | 1.24(1.18-1.31) | <0.001 |  | 0.214 | 1.24(1.18-1.30) | <0.001 |
| rs11568818 | C | T | 0.024 | 1.02(0.98-1.07) | 0.247 |  | 0.034 | 1.03(1.00-1.07) | 0.058 |
| rs8102476 | C | T | 0.087 | 1.09(1.05-1.14) | <0.001 |  | 0.094 | 1.10(1.06-1.14) | <0.001 |
| rs11672691 | A | G | 0.035 | 1.04(0.99-1.08) | 0.097 |  | 0.043 | 1.04(1.00-1.09) | 0.037 |
| rs1058205 | T | C | 0.145 | 1.16(1.10-1.21) | <0.001 |  | 0.136 | 1.15(1.09-1.20) | <0.001 |
| rs2292884 | G | A | 0.074 | 1.08(1.03-1.13) | 0.001 |  | 0.086 | 1.09(1.04-1.14) | <0.001 |
| rs7611694 | A | C | 0.077 | 1.08(1.04-1.12) | <0.001 |  | 0.077 | 1.08(1.04-1.12) | <0.001 |
| rs7758229 | T | G | 0.093 | 1.10(1.05-1.14) | <0.001 |  | 0.082 | 1.09(1.04-1.13) | <0.001 |
| rs10993994 | T | C | 0.192 | 1.21(1.17-1.26) | <0.001 |  | 0.185 | 1.20(1.16-1.25) | <0.001 |
| rs4430796 | A | G | 0.138 | 1.15(1.10-1.20) | <0.001 |  | 0.108 | 1.11(1.07-1.15) | <0.001 |
| rs8064454 | C | A | 0.144 | 1.15(1.11-1.20) | <0.001 |  | 0.170 | 1.19(1.14-1.23) | <0.001 |

*, beta was the log OR of per-allele index SNP with outcome from univariate logistics regression.


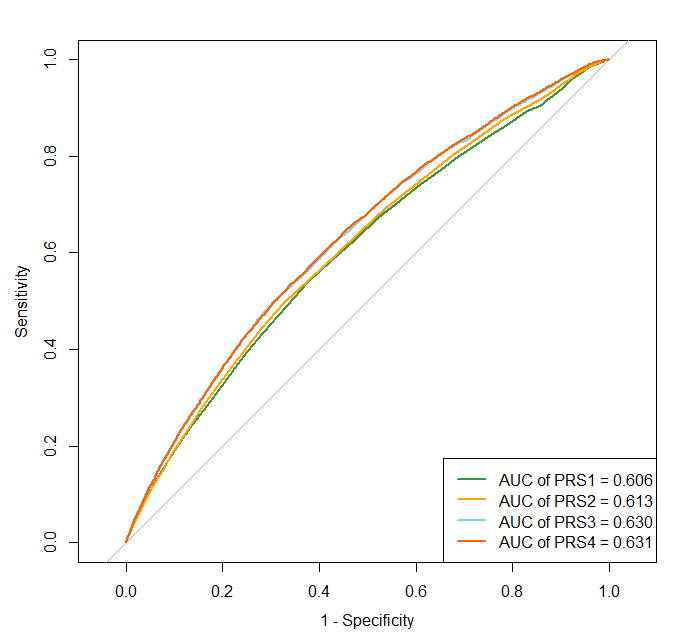


**S2 Fig. Accuracy of prostate cancer predicted by different polygenic risk scores (PRS).**

Unweighted PRS1 and PRS2 were calculated as the sum of number of risk allele from all selected SNPs and SNPs significantly associated with prostate cancer. Weighted PRS3 and PRS4 were calculated as the sum of risk allele from all selected SNPs and validated SNPs weighted with beta of SNP from univariate logistics regression.

## S5 Table. Baseline characteristics associated with the incidence of prostate cancer.

| Subgroups ^a^ | Participants,  No. (%) | PC cases,  No. (%) | Follow-up,  1000 PYs | IR of PC,  per 1000 PYs | P value for  K-M curve | Adjusted  HR (95%CI) ^b^ | P value ^b^ |
| --- | --- | --- | --- | --- | --- | --- | --- |
| Age at entrance |  | |  |  | |  |  |
| <60 years | 17535(35.1) | 1507(25.2) | 192.78 | 7.82 | <0.001 | Ref. |  |
| 60-64 years | 15929(31.9) | 2089(34.9) | 185.48 | 11.26 |  | 1.39(1.27-1.51) | <0.001 |
| 65-69 years | 10934(21.9) | 1607(26.8) | 123.59 | 13.00 |  | 1.60(1.46-1.75) | <0.001 |
| ≥ 70 years | 5509(11.0) | 783(13.1) | 59.11 | 13.25 |  | 1.59(1.42-1.78) | <0.001 |
| Race |  |  |  |  |  |  |  |
| White | 45021(90.2) | 5419(90.5) | 508.35 | 10.66 | <0.001 | Ref. |  |
| Black | 1826(3.7) | 298(5.0) | 17.98 | 16.58 |  | 1.53(1.32-1.77) | <0.001 |
| Other | 3030 (6.1) | 268(4.5) | 34.33 | 7.81 |  | 0.69(0.59-0.82) | <0.001 |
| Body mass index |  | |  |  |  |  |  |
| 0-25 kg/m^2^ | 12767(25.6) | 1641(27.4) | 144.96 | 11.32 | 0.001 | Ref. |  |
| 25-30 kg/m^2^ | 25165 (50.4) | 3075(51.4) | 284.04 | 10.83 |  | 0.99(0.91-1.07) | 0.734 |
| >30 kg/m^2^ | 11280(22.6) | 1174(19.6) | 124.15 | 9.46 |  | 0.90(0.82-0.99) | 0.041 |
| Smoking state |  |  |  |  |  |  |  |
| Never | 18908(37.9) | 2462(41.1) | 216.02 | 11.40 | <0.001 | Ref. |  |
| Current | 5095(10.2) | 516(8.6) | 53.73 | 9.60 |  | 0.87(0.77-0.98) | 0.021 |
| Former | 25893(51.9) | 3006(50.2) | 291.06 | 10.33 |  | 0.90(0.84-0.96) | 0.002 |
| Family history of prostate cancer | | |  |  | |  |  |
| No | 45021(90.22) | 5197(86.8) | 507.30 | 10.24 | <0.001 | Ref. |  |
| Yes | 659(7.4) | 659(11.0) | 40.18 | 16.40 |  | 1.52(1.37-1.69) | <0.001 |
| History of previous PSA screening | | |  |  | |  |  |
| No | 22710(45.5) | 2570(42.9) | 250.98 | 10.24 | <0.001 | Ref. |  |
| 1 time | 18383(36.8) | 2149(35.9) | 207.17 | 10.37 |  | 0.96(0.89-1.04) | 0.289 |
| ≥ 2 times | 8812(17.7) | 1266(21.1) | 102.78 | 12.32 |  | 1.09(0.99-1.19) | 0.060 |
| Enlarged prostate |  |  |  |  |  |  |  |
| No | 40173(80.5) | 4554(76.1) | 451.17 | 10.09 | <0.001 | Ref. |  |
| Yes | 9667(19.4) | 1423(23.8) | 108.96 | 13.06 |  | 1.18(1.09-1.27) | <0.001 |
| History of diabetes |  |  |  |  |  |  |  |
| No | 45731(91.6) | 5624(94.0) | 515.54 | 10.91 | <0.001 | Ref. |  |
| Yes | 3915(7.8) | 331(5.5) | 42.38 | 7.81 |  | 0.65(0.56-0.75) | <0.001 |

PC, prostate cancer; PY, person-year; IR, incidence rate.

^a^Missing data in the index variable were not shown.

^b^adjusted all index variables listed in the table, and missing data of each variable were coded as independent group in the multivariable COX regression.

## S6 Table. Accuracy of 10-year incidence risk of prostate cancer predicted by different polygenic risk scores (PRS).

| PRS | Mean | Std.  Error | Median | Interquartile  Range | Minimum | Maximum | Area Under the Curve | | | |
| --- | --- | --- | --- | --- | --- | --- | --- | --- | --- | --- |
|  |  |  |  |  |  |  | Area | LCI | UCI | P value |
| PRS1 | 88.97 | 0.045 | 90.00 | 12.00 | 48.00 | 121.00 | 0.606 | 0.559 | 0.614 | Ref. |
| PRS2 | 69.76 | 0.038 | 71.00 | 11.00 | 32.00 | 98.00 | 0.613 | 0.606 | 0.622 | <0.001 |
| PRS3 | 9.09 | 0.005 | 9.17 | 1.20 | 2.98 | 13.11 | 0.630 | 0.623 | 0.638 | <0.001 |
| PRS4 | 8.65 | 0.004 | 8.71 | 1.17 | 2.68 | 12.68 | 0.631 | 0.624 | 0.639 | <0.001 |

AUCs were compared with DeLong test.

**S7 Table. Differences in incidence and mortality of prostate cancer in different genetic risk groups.**

| Subgroups | Participants,  No. (%) | Event,  No. (%) | Follow-up,  1000 PYs | Event rate,  per 1000 PYs | Unadjusted,  HR (95%CI) | P  value | Adjusted,  HR (95%CI) ^a^ | P  value ^a^ |
| --- | --- | --- | --- | --- | --- | --- | --- | --- |
| PCa incidence | 49907 |  |  |  |  |  |  |  |
| Low PRS | 31717(63.6) | 2786(46.5) | 365.53 | 7.62 | Ref. | <0.001 | Ref. |  |
| High PRS | 18190(36.4) | 3200(53.5) | 195.42 | 16.38 | 2.15(2.04-2.26) |  | 2.10(2.00-2.21) | <0.001 |
| PCa mortality |  |  |  |  |  |  |  |  |
| Low PRS | 31717(63.6) | 251(48.6) | 575.26 | 0.44 | Ref. | <0.001 | Ref. |  |
| High PRS | 18190(36.4) | 265(51.4) | 329.34 | 0.80 | 1.85(1.55-2.19) |  | 1.81(1.51-2.16) | <0.001 |

^a^, adjusted available variables associated with prostate cancer listed in S5 Table, and missing data of each variable were coded as independent group in the multivariable COX regression.

## S8 Table. Bootstrap resampling analyses on overall and age-specific cut-off values of PSA screening for PCa with 2000 iterations by genetic risks.

| Cut-off value | Participants | Events | Optimal value | 95%CI |
| --- | --- | --- | --- | --- |
| PSA screening cut-off value for low PRS | 12210/1295 | 375/1295 | 1.44 | 1.37-1.62 |
| PSA screening cut-off value for high PRS | 6345/4095 | 385/1521 | 1.62 | 1.43-1.82 |
| Age-specific PSA screening cutoff values | | | | |
| Total population |  |  |  |  |
| <60 years | 7161/3122 | 207/717 | 1.41 | 1.23-2.29 |
| 60-64 years | 5899/3468 | 269/974 | 1.46 | 1.36-1.77 |
| 65-69 years | 3654/2706 | 163/788 | 1.59 | 1.53-1.85 |
| ≥ 70 years | 2096/1139 | 112/346 | 2.19 | 1.72-2.92 |
| Low PRS |  |  |  |  |
| <60 years | 4654/1941 | 111/337 | 1.34 | 1.12-1.45 |
| 60-64 years | 4259/1763 | 167/408 | 1.62 | 1.23-1.85 |
| 65-69 years | 2512/1553 | 88/341 | 1.60 | 1.45-1.84 |
| ≥ 70 years | 1430/693 | 62/156 | 2.01 | 1.69-2.58 |
| High PRS |  |  |  |  |
| <60 years | 2598/1090 | 117/359 | 1.60 | 1.39-1.83 |
| 60-64 years | 1920/1425 | 132/536 | 1.48 | 1.34-2.01 |
| 65-69 years | 1291/1004 | 108/414 | 1.84 | 1.56-2.15 |
| ≥ 70 years | 751/361 | 67/173 | 2.68 | 1.82-3.00 |

95%CI, 95% confidential interval.

References:

[1]. Gudmundsson, J., et al., Two variants on chromosome 17 confer prostate cancer risk, and the one in TCF2 protects against type 2 diabetes. Nat Genet, 2007. 39(8): p. 977-83.

[2]. Murabito, J.M., et al., A genome-wide association study of breast and prostate cancer in the NHLBI's Framingham Heart Study. BMC Med Genet, 2007. 8 Suppl 1(Suppl 1): p. S6.

[3]. Thomas, G., et al., Multiple loci identified in a genome-wide association study of prostate cancer. Nat Genet, 2008. 40(3): p. 310-5.

[4]. Eeles, R.A., et al., Multiple newly identified loci associated with prostate cancer susceptibility. Nat Genet, 2008. 40(3): p. 316-21.

[5]. Gudmundsson, J., et al., Common sequence variants on 2p15 and Xp11.22 confer susceptibility to prostate cancer. Nat Genet, 2008. 40(3): p. 281-3.

[6]. Sun, J., et al., Sequence variants at 22q13 are associated with prostate cancer risk. Cancer Res, 2009. 69(1): p. 10-5.

[7]. Eeles, R.A., et al., Identification of seven new prostate cancer susceptibility loci through a genome-wide association study. Nat Genet, 2009. 41(10): p. 1116-21.

[8]. Gudmundsson, J., et al., Genome-wide association and replication studies identify four variants associated with prostate cancer susceptibility. Nat Genet, 2009. 41(10): p. 1122-6.

[9]. Takata, R., et al., Genome-wide association study identifies five new susceptibility loci for prostate cancer in the Japanese population. Nat Genet, 2010. 42(9): p. 751-4.

[10]. Haiman, C.A., et al., Genome-wide association study of prostate cancer in men of African ancestry identifies a susceptibility locus at 17q21. Nat Genet, 2011. 43(6): p. 570-3.

[11]. Schumacher, F.R., et al., Genome-wide association study identifies new prostate cancer susceptibility loci. Hum Mol Genet, 2011. 20(19): p. 3867-75.

[12]. Stacey, S.N., et al., A germline variant in the TP53 polyadenylation signal confers cancer susceptibility. Nat Genet, 2011. 43(11): p. 1098-103.

[13]. Nam, R.K., et al., New variants at 10q26 and 15q21 are associated with aggressive prostate cancer in a genome-wide association study from a prostate biopsy screening cohort. Cancer Biol Ther, 2011. 12(11): p. 997-1004.

[14]. Cheng, I., et al., Evaluating genetic risk for prostate cancer among Japanese and Latinos. Cancer Epidemiol Biomarkers Prev, 2012. 21(11): p. 2048-58.

[15]. Xu, J., et al., Genome-wide association study in Chinese men identifies two new prostate cancer risk loci at 9q31.2 and 19q13.4. Nat Genet, 2012. 44(11): p. 1231-5.

[16]. Amin, A.O.A., et al., A meta-analysis of genome-wide association studies to identify prostate cancer susceptibility loci associated with aggressive and non-aggressive disease. Hum Mol Genet, 2013. 22(2): p. 408-15.

[17]. Gudmundsson, J., et al., A study based on whole-genome sequencing yields a rare variant at 8q24 associated with prostate cancer. Nat Genet, 2012. 44(12): p. 1326-9.

[18]. Eeles, R.A., et al., Identification of 23 new prostate cancer susceptibility loci using the iCOGS custom genotyping array. Nat Genet, 2013. 45(4): p. 385-91, 391e1-2.

[19]. Kote-Jarai, Z., et al., Fine-mapping identifies multiple prostate cancer risk loci at 5p15, one of which associates with TERT expression. Hum Mol Genet, 2013. 22(12): p. 2520-8.

[20]. Lange, E.M., et al., Genome-wide association scan for variants associated with early-onset prostate cancer. PLoS One, 2014. 9(4): p. e93436.

[21]. Al, O.A., et al., A meta-analysis of 87,040 individuals identifies 23 new susceptibility loci for prostate cancer. Nat Genet, 2014. 46(10): p. 1103-9.

[22]. Stegeman, S., et al., A Large-Scale Analysis of Genetic Variants within Putative miRNA Binding Sites in Prostate Cancer. Cancer Discov, 2015. 5(4): p. 368-79.

[23]. Berndt, S.I., et al., Two susceptibility loci identified for prostate cancer aggressiveness. Nat Commun, 2015. 6: p. 6889.

[24]. Hoffmann, T.J., et al., A large multiethnic genome-wide association study of prostate cancer identifies novel risk variants and substantial ethnic differences. Cancer Discov, 2015. 5(8): p. 878-91.

[25]. Wang, M., et al., Large-scale association analysis in Asians identifies new susceptibility loci for prostate cancer. Nat Commun, 2015. 6: p. 8469.

[26]. Hoffmann, T.J., et al., Genome-wide association study of prostate-specific antigen levels identifies novel loci independent of prostate cancer. Nat Commun, 2017. 8: p. 14248.

[27]. Gomez-Acebo, I., et al., Risk Model for Prostate Cancer Using Environmental and Genetic Factors in the Spanish Multi-Case-Control (MCC) Study. Sci Rep, 2017. 7(1): p. 8994.

[28]. Du Z, et al., Genetic risk of prostate cancer in Ugandan men. Prostate, 2018. 78(5): p. 370-376.

[29]. Schumacher, F.R., et al., Association analyses of more than 140,000 men identify 63 new prostate cancer susceptibility loci. Nat Genet, 2018. 50(7): p. 928-936.

[30]. Takata, R., et al., 12 new susceptibility loci for prostate cancer identified by genome-wide association study in Japanese population. Nat Commun, 2019. 10(1): p. 4422.

[31]. Ray, D. and N. Chatterjee, A powerful method for pleiotropic analysis under composite null hypothesis identifies novel shared loci between Type 2 Diabetes and Prostate Cancer. PLOS Genetics, 2020. 16(12): p. e1009218.

[32]. Conti, D.V., et al., Trans-ancestry genome-wide association meta-analysis of prostate cancer identifies new susceptibility loci and informs genetic risk prediction. Nat Genet, 2021. 53(1): p. 65-75.

[33]. Sipeky, C., et al., Novel prostate cancer susceptibility gene SP6 predisposes patients to aggressive disease. Prostate Cancer Prostatic Dis, 2021. 24(4): p. 1158-1166.

[34]. Sakaue, S., et al., A cross-population atlas of genetic associations for 220 human phenotypes. Nat Genet, 2021. 53(10): p. 1415-1424.

[35]. Nazarian, A., et al., Genome-wide analysis of genetic predisposition to common polygenic cancers. J Appl Genet, 2022. 63(2): p. 315-325.
